# Supplementary figures and images for: Mapping cannabis potency in medical and recreational programs in the United States
Source: PLoS One. 2020 Mar 26;15(3):e0230167. doi: 10.1371/journal.pone.0230167 (PMC7098613; doi:10.1371/journal.pone.0230167)

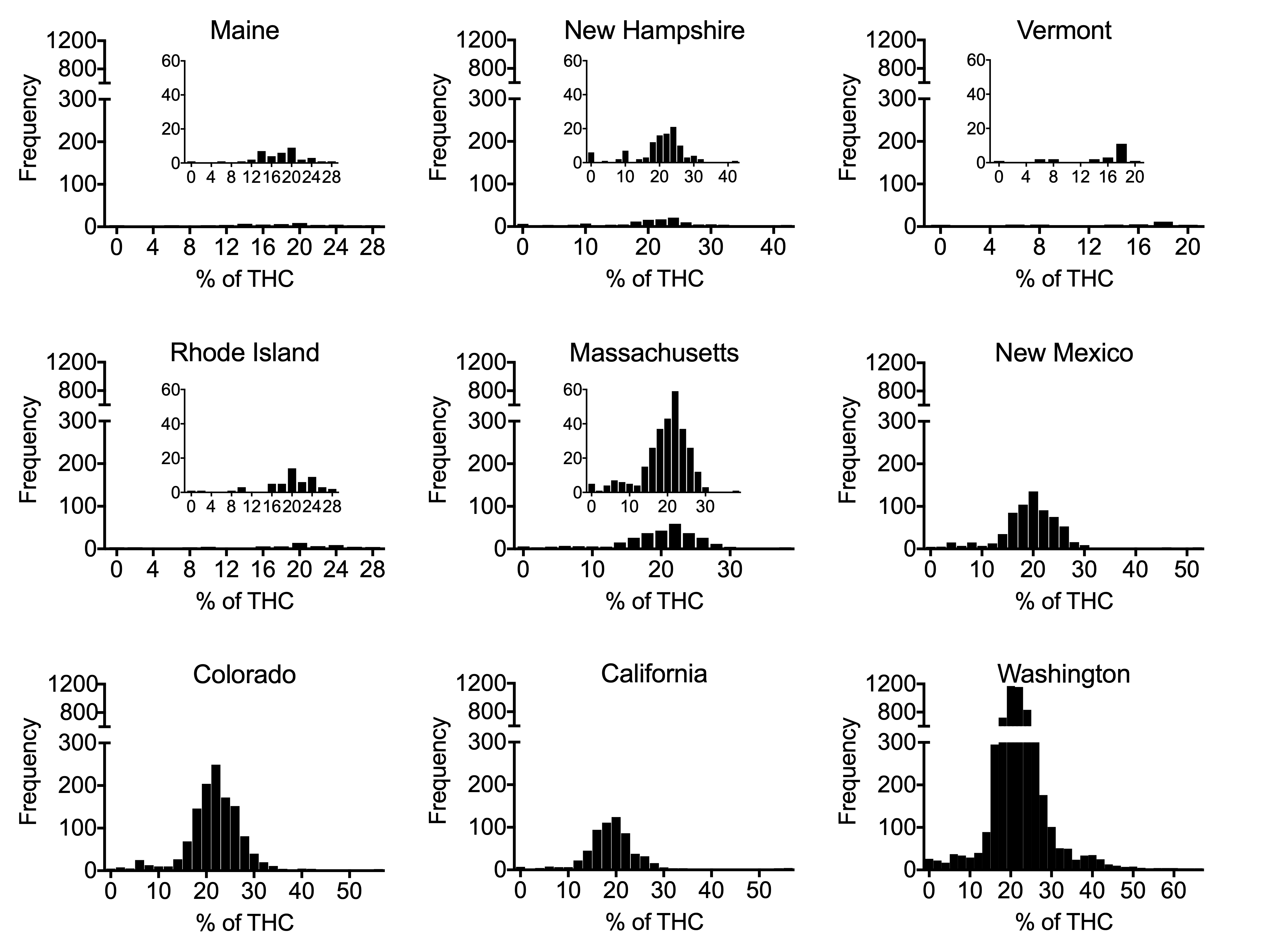

Supplement: S1 Fig — Abundance (frequency) of products in relation of given THC contents (potencies). Note that X axis varies among states. Y axes are similar in all graphs, however inset graphs show smaller Y axes’ scales to better represent data from states with less abundance products. (TIFF) [file pone.0230167.s001.tiff]

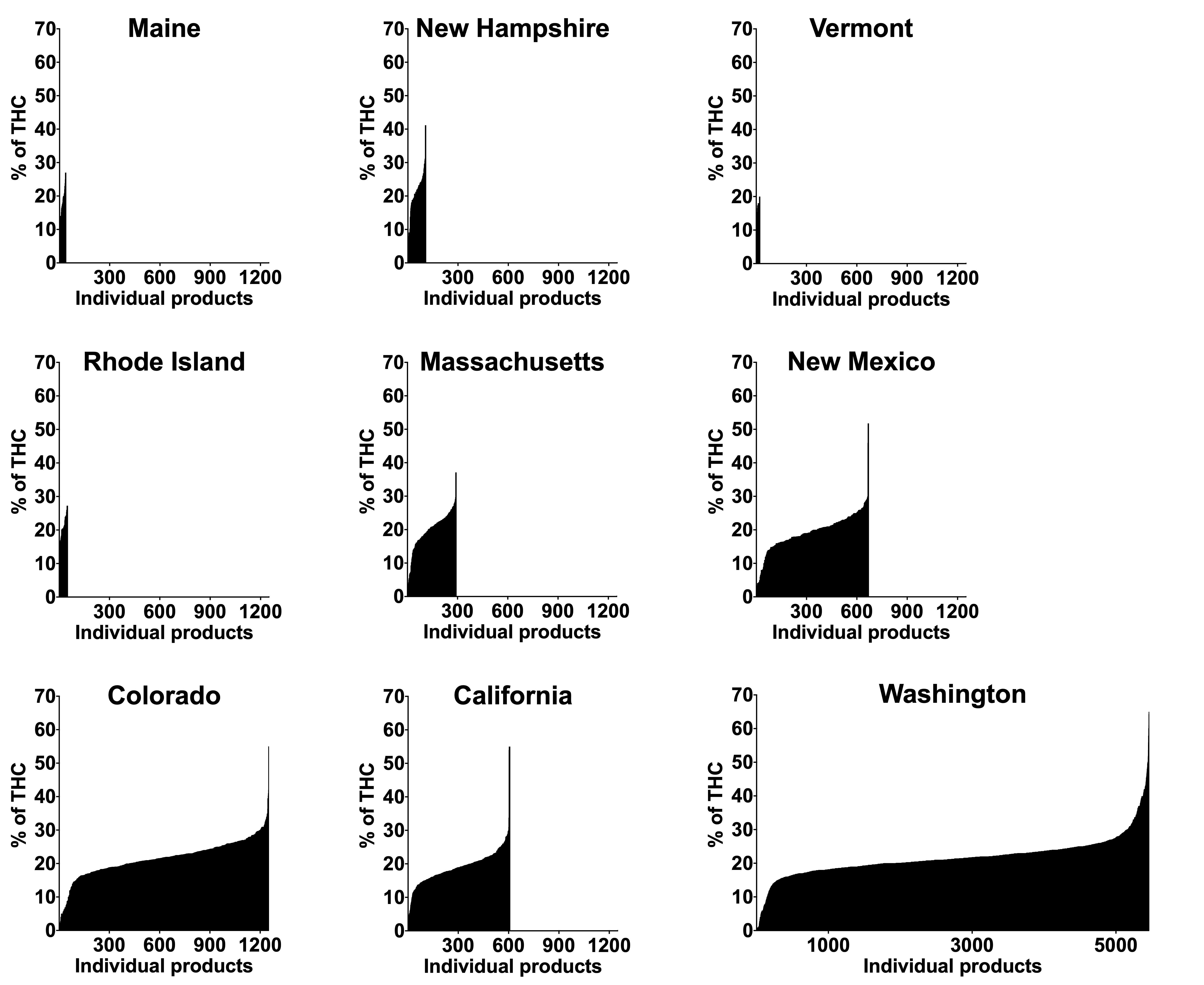

Supplement: S2 Fig — Every individual product offered in a given state is plotted on the X axis and its THC content is shown on the Y axis. Note that Washington state is presented with a larger X axis scale since this state offers many more products than the other surveyed states. (TIFF) [file pone.0230167.s002.tiff]

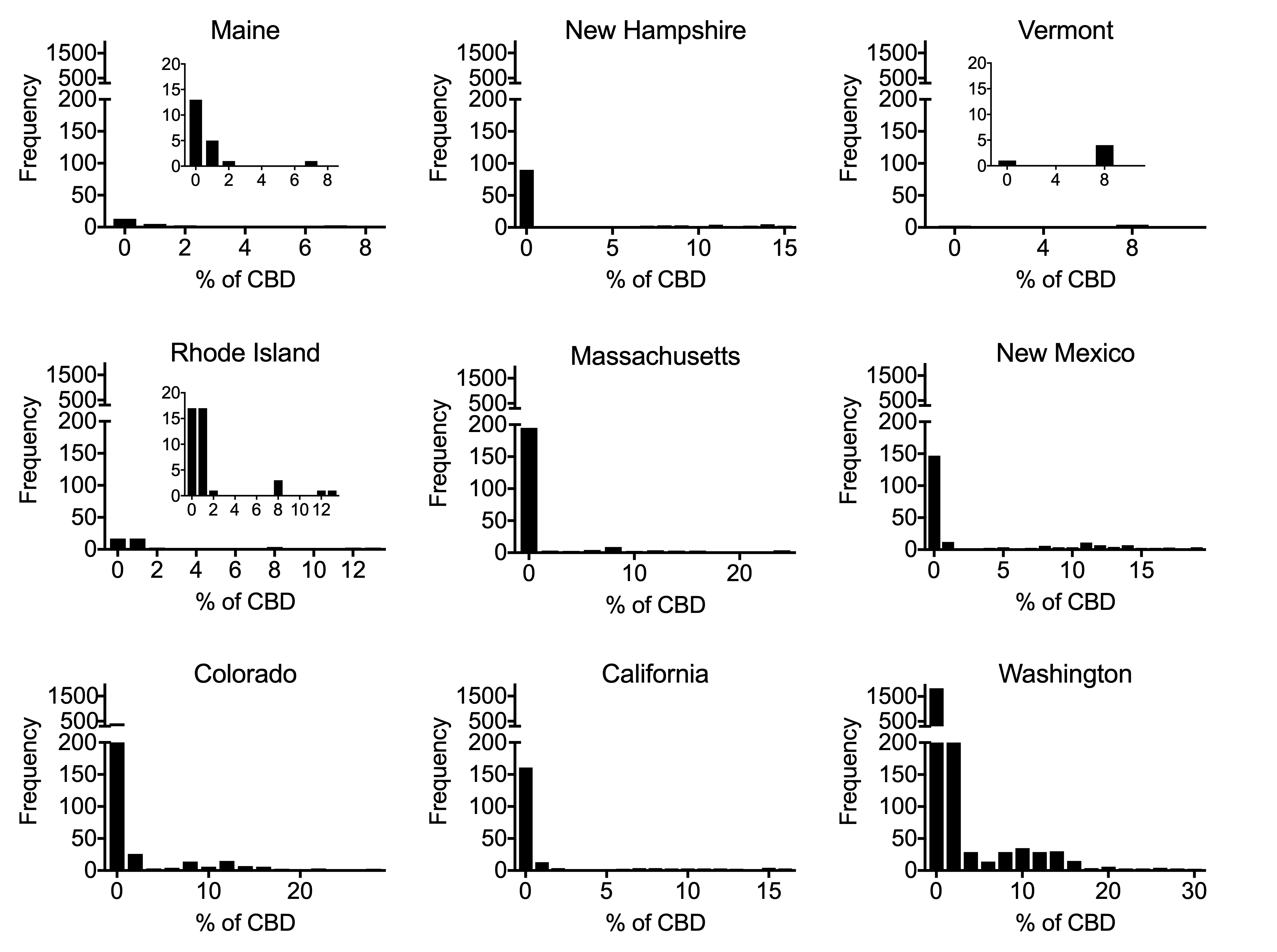

Supplement: S3 Fig — Abundance (frequency) of products in relation of given CBD contents. Note that X axis varies among states. Y axes are similar in all graphs, however inset graphs show smaller Y axes’ scales to better represent data from states with less abundance products. (TIFF) [file pone.0230167.s003.tiff]

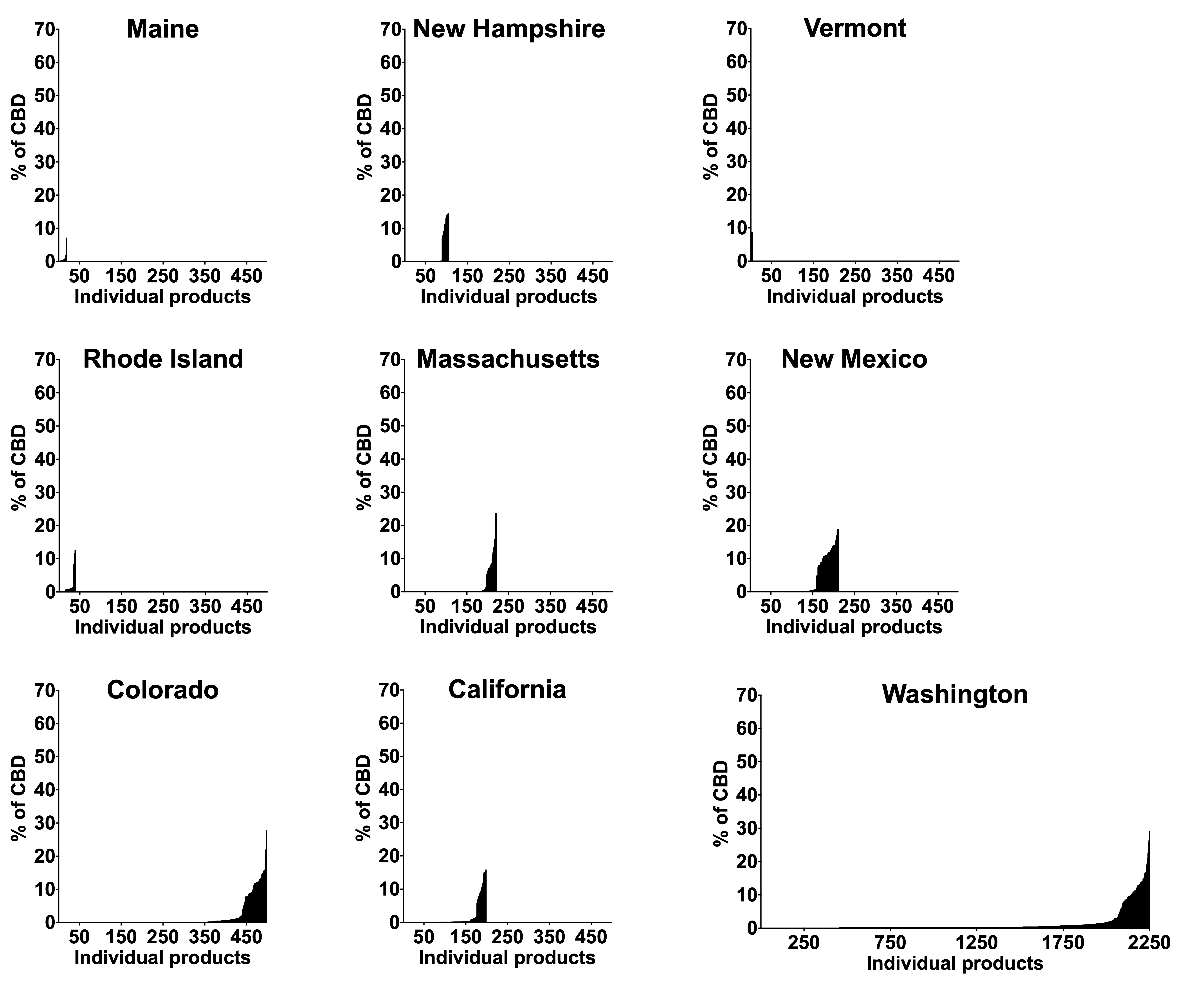

Supplement: S4 Fig — Every individual product offered in a given state is plotted on the X axis and its CBD content is shown on the Y axis. Note that Washington state is presented with a larger X axis scale since this state offers many more products than the other surveyed states. In most states, the vast majority of products have 0% CBD, as shown on X axis towards the left. (TIFF) [file pone.0230167.s004.tiff]
