# Supplementary material for: Mapping cannabis potency in medical and recreational programs in the United States
Source: PLoS One. 2020 Mar 26;15(3):e0230167. doi: 10.1371/journal.pone.0230167 (PMC7098613; doi:10.1371/journal.pone.0230167)
Supplement: S1 Table — (DOCX) [file pone.0230167.s005.docx]

**S1 Table. Legalized United States Recreational Programs**

| **State** | **Pain as Qualifying Condition? (Y/N)** | **Licensed Dispensaries as of August 2018 (N)** | **Dispensaries Sampled (N)** | **Dispensaries with Online Presence (N)** | **Sampled Dispensaries with THC data available (N)** |
| --- | --- | --- | --- | --- | --- |
| Alaska | Yes | 53 | 20 | 18 | 1 |
| California | Yes | 335 | 268 | 210 | 26 |
| Colorado | Yes | >520 | 200 | 88 | 44 |
| Nevada | Yes | 60 | 60 | 55 | 22 |
| Oregon | Yes | 560 | Not sampled |  |  |
| Washington | Yes | >575 | 80 | 54 | 26 |
| Washington D.C. | No | 6 | 6 | 6 | 4 |
