# Supplementary material for: Mapping cannabis potency in medical and recreational programs in the United States
Source: PLoS One. 2020 Mar 26;15(3):e0230167. doi: 10.1371/journal.pone.0230167 (PMC7098613; doi:10.1371/journal.pone.0230167)
Supplement: S2 Table — a At time of data collection, cannabis recently legalized for medical purposes but did not have any active and open dispensaries. b The two dispensaries in Delaware exist under the same license. Only one website exists for both locations. c At time of data collection, there was a hold on Maryland cannabis practices. Therefore, no data was collected in Maryland.d Shortly after data collection, Massachusetts legalized cannabis for recreational purposes. For the purposes of this study, MA was considered a medical-only state. (DOCX) [file pone.0230167.s006.docx]

**S2 Table. Legalized United Stated Medical Programs**

| **State** | **Pain as Qualifying Condition? (Y/N)** | **Licensed Dispensaries as of May 2018 (N)** | **Dispensaries Sampled (N)** | **Dispensaries with Online Presence (N)** | **Sampled Dispensaries with THC data available (N)** |
| --- | --- | --- | --- | --- | --- |
| Arizona | Yes | >100 | 20 | 16 | 2 |
| Arkansas^a^ | Yes | 0 |  |  |  |
| Connecticut | Yes | 9 | 9 | 9 | 9 |
| Delaware | Yes | 2 | 2 | 1^b^ | 0 |
| Florida | Yes | 32 | 32 | 29 | 24 |
| Hawaii | Yes | 8 | 8 | 7 | 2 |
| Illinois | Yes | 54 | 54 | 53 | 23 |
| Maine | Yes | 8 | 8 | 7 | 5 |
| Maryland^c^ | Yes | 30 | Not sampled |  |  |
| Massachusetts^d^ | Yes | 20 | 20 | 20 | 18 |
| Michigan | Yes | > 100 | 100 | 100 | 4 |
| Minnesota | Yes | 8 | 8 | 8 | 8 |
| Montana | Yes | >50 | 30 | 26 | 3 |
| New Hampshire | Yes | 4 | 4 | 4 | 4 |
| New Jersey | Yes | 5 | 5 | 5 | 0 |
| New Mexico | Yes | 72 | 72 | 67 | 44 |
| New York | No | 19 | 19 | 19 | 4 |
| North Dakota | Yes | 0* |  |  |  |
| Ohio | Yes | 0* |  |  |  |
| Pennsylvania | Yes | 13 | 13 | 13 | 1 |
| Rhode Island | Yes | 3 | 3 | 3 | 3 |
| West Virginia | Yes | 0* |  |  |  |
| Vermont | Yes | 5 | 5 | 5 | 3 |

^a^ At time of data collection, cannabis recently legalized for medical purposes but did not have any active and open dispensaries.

^b^ The two dispensaries in Delaware exist under the same license. Only one website exists for both locations.

^c^ At time of data collection, there was a hold on Maryland cannabis practices. Therefore, no data was collected in Maryland.

^d^ Shortly after data collection, Massachusetts legalized cannabis for recreational purposes. For the purposes of this study, MA was considered a medical-only state.
