# Supplementary material for: Mapping cannabis potency in medical and recreational programs in the United States
Source: PLoS One. 2020 Mar 26;15(3):e0230167. doi: 10.1371/journal.pone.0230167 (PMC7098613; doi:10.1371/journal.pone.0230167)
Supplement: S3 Table — (DOCX) [file pone.0230167.s007.docx]

**S3 Table**. **Descriptive statistics for THC (%, top) and CBD concentrations (%, bottom) in all products offered in each sampled state.**

| % THC |  |  |  |  |  |  |  |  |  |
| --- | --- | --- | --- | --- | --- | --- | --- | --- | --- |
|  | ME | NH | VT | RI | MA | NM | CO | WA | CA |
| 25% Percentile | 14.04 | 18.61 | 14 | 17.87 | 17 | 16.17 | 18.93 | 19 | 16.18 |
| Median | 17.59 | 21.49 | 17.36 | 20.46 | 20.81 | 19 | 21.85 | 21.3 | 19 |
| 75% Percentile | 20.26 | 24.28 | 18 | 23.91 | 23.1 | 21.59 | 24.94 | 23.9 | 21.56 |
| Mean | 17.56 | 20.27 | 15.23 | 19.88 | 19.38 | 18.98 | 21.54 | 21.71 | 18.93 |
| Std. Deviation | 4.389 | 7.173 | 4.301 | 5.182 | 6.011 | 5.606 | 5.606 | 5.892 | 5.398 |
| Std. Error of Mean | 0.7216 | 0.6967 | 0.9385 | 0.7402 | 0.3299 | 0.2274 | 0.1582 | 0.07973 | 0.2193 |
|  |  |  |  |  |  |  |  |  |  |
| % CBD |  |  |  |  |  |  |  |  |  |
|  | ME | NH | VT | RI | MA | NM | CO | WA | CA |
| 25% Percentile | 0.2 | 0.05 | 8 | 0.047 | 0 | 0 | 0 | 0.09 | 0 |
| Median | 0.4 | 0.05 | 8.345 | 0.851 | 0.1 | 0.06 | 0 | 0.2 | 0.04 |
| 75% Percentile | 0.84 | 0.09 | 8.69 | 1.221 | 0.1 | 3.53 | 0.5 | 0.655 | 0.2325 |
| Mean | 0.8621 | 1.806 | 8.345 | 1.772 | 1.321 | 2.947 | 1.461 | 1.324 | 1.34 |
| Std. Deviation | 1.603 | 4.259 | 0.3984 | 3.279 | 3.951 | 5.228 | 3.918 | 3.476 | 3.518 |
| Std. Error of Mean | 0.3677 | 0.4156 | 0.1992 | 0.5251 | 0.264 | 0.3599 | 0.1756 | 0.07329 | 0.25 |
