# Supplementary material for: Mapping cannabis potency in medical and recreational programs in the United States
Source: PLoS One. 2020 Mar 26;15(3):e0230167. doi: 10.1371/journal.pone.0230167 (PMC7098613; doi:10.1371/journal.pone.0230167)
Supplement: S4 Table — One way-ANOVA followed by Turkey’s multiple comparisons test was used, and P values are reported. A P<0.05 was considered statistically significant. ns = not statistically significant. (DOCX) [file pone.0230167.s008.docx]

**S4 Table**. **Comparisons of THC concentrations (%) in all products between each sampled state.** One way-ANOVA followed by Turkey’s multiple comparisons test was used, and P values are reported. A P<0.05 was considered statistically significant.

| Number of families | 1 |  |  |  |  |
| --- | --- | --- | --- | --- | --- |
| Number of comparisons per family | 36 |  |  |  |  |
| Alpha | 0.05 |  |  |  |  |
| Df | 8468 |  |  |  |  |
| Tukey's multiple comparisons test | Mean Diff. | 95.00% CI of diff. | Significant? | Summary | Adjusted P Value |
| ME vs. NH | -2.703 | -6.143 to 0.7362 | No | ns | 0.2631 |
| ME vs. VT | 2.338 | -2.584 to 7.259 | No | ns | 0.868 |
| ME vs. RI | -2.312 | -6.235 to 1.611 | No | ns | 0.6632 |
| ME vs. MA | -1.819 | -4.941 to 1.303 | No | ns | 0.6773 |
| ME vs. NM | -1.417 | -4.468 to 1.633 | No | ns | 0.8817 |
| ME vs. CO | -3.98 | -6.985 to -0.9757 | Yes | ** | 0.0013 |
| ME vs. WA | -4.144 | -7.115 to -1.172 | Yes | *** | 0.0005 |
| ME vs. CA | -1.369 | -4.42 to 1.681 | No | ns | 0.901 |
| NH vs. VT | 5.041 | 0.7384 to 9.344 | Yes | ** | 0.0085 |
| NH vs. RI | 0.3914 | -2.72 to 3.503 | No | ns | >0.9999 |
| NH vs. MA | 0.8846 | -1.125 to 2.894 | No | ns | 0.9107 |
| NH vs. NM | 1.286 | -0.6099 to 3.182 | No | ns | 0.4701 |
| NH vs. CO | -1.277 | -3.099 to 0.545 | No | ns | 0.4225 |
| NH vs. WA | -1.44 | -3.207 to 0.3261 | No | ns | 0.2177 |
| NH vs. CA | 1.334 | -0.5622 to 3.231 | No | ns | 0.417 |
| VT vs. RI | -4.65 | -9.348 to 0.04865 | No | ns | 0.055 |
| VT vs. MA | -4.157 | -8.21 to -0.1032 | Yes | * | 0.0394 |
| VT vs. NM | -3.755 | -7.753 to 0.2433 | No | ns | 0.0855 |
| VT vs. CO | -6.318 | -10.28 to -2.354 | Yes | **** | <0.0001 |
| VT vs. WA | -6.481 | -10.42 to -2.543 | Yes | **** | <0.0001 |
| VT vs. CA | -3.707 | -7.705 to 0.2916 | No | ns | 0.0946 |
| RI vs. MA | 0.4931 | -2.264 to 3.25 | No | ns | 0.9998 |
| RI vs. NM | 0.8947 | -1.78 to 3.57 | No | ns | 0.9822 |
| RI vs. CO | -1.668 | -4.291 to 0.9547 | No | ns | 0.5622 |
| RI vs. WA | -1.832 | -4.417 to 0.7531 | No | ns | 0.4065 |
| RI vs. CA | 0.9428 | -1.733 to 3.618 | No | ns | 0.9753 |
| MA vs. NM | 0.4016 | -0.8277 to 1.631 | No | ns | 0.9847 |
| MA vs. CO | -2.162 | -3.273 to -1.05 | Yes | **** | <0.0001 |
| MA vs. WA | -2.325 | -3.343 to -1.307 | Yes | **** | <0.0001 |
| MA vs. CA | 0.4497 | -0.7803 to 1.68 | No | ns | 0.9691 |
| NM vs. CO | -2.563 | -3.453 to -1.673 | Yes | **** | <0.0001 |
| NM vs. WA | -2.727 | -3.497 to -1.956 | Yes | **** | <0.0001 |
| NM vs. CA | 0.04812 | -0.9859 to 1.082 | No | ns | >0.9999 |
| CO vs. WA | -0.1634 | -0.7271 to 0.4003 | No | ns | 0.9931 |
| CO vs. CA | 2.611 | 1.72 to 3.502 | Yes | **** | <0.0001 |
| WA vs. CA | 2.775 | 2.003 to 3.546 | Yes | **** | <0.0001 |

ns = not statistically significant.
