# Supplementary material for: Mapping cannabis potency in medical and recreational programs in the United States
Source: PLoS One. 2020 Mar 26;15(3):e0230167. doi: 10.1371/journal.pone.0230167 (PMC7098613; doi:10.1371/journal.pone.0230167)
Supplement: S5 Table — One way-ANOVA followed by Turkey’s multiple comparisons test was used, and P values are reported. A P<0.05 was considered statistically significant. ns = not statistically significant. (DOCX) [file pone.0230167.s009.docx]

**S5 Table. Comparisons of CBD concentrations (%) in all products between each sampled state.** One way-ANOVA followed by Turkey’s multiple comparisons test was used, and P values are reported. A P<0.05 was considered statistically significant.

| Number of families | 1 |  |  |  |  |
| --- | --- | --- | --- | --- | --- |
| Number of comparisons per family | 36 |  |  |  |  |
| Alpha | 0.05 |  |  |  |  |
| Df | 3538 |  |  |  |  |
|  |  |  |  |  |  |
| Tukey's multiple comparisons test | Mean Diff. | 95.00% CI of diff. | Significant? | Summary | Adjusted P Value |
| ME vs. NH | -0.9441 | -3.817 to 1.929 | No | ns | 0.9841 |
| ME vs. VT | -7.483 | -13.82 to -1.143 | Yes | ** | 0.0078 |
| ME vs. RI | -0.9101 | -4.134 to 2.314 | No | ns | 0.9942 |
| ME vs. MA | -0.4591 | -3.213 to 2.295 | No | ns | 0.9999 |
| ME vs. NM | -2.085 | -4.845 to 0.6758 | No | ns | 0.316 |
| ME vs. CO | -0.5985 | -3.292 to 2.095 | No | ns | 0.9989 |
| ME vs. WA | -0.462 | -3.117 to 2.193 | No | ns | 0.9998 |
| ME vs. CA | -0.4779 | -3.246 to 2.29 | No | ns | 0.9998 |
| NH vs. VT | -6.539 | -12.41 to -0.6677 | Yes | * | 0.0162 |
| NH vs. RI | 0.03401 | -2.127 to 2.195 | No | ns | >0.9999 |
| NH vs. MA | 0.485 | -0.8781 to 1.848 | No | ns | 0.9737 |
| NH vs. NM | -1.141 | -2.517 to 0.2358 | No | ns | 0.1989 |
| NH vs. CO | 0.3455 | -0.8921 to 1.583 | No | ns | 0.9946 |
| NH vs. WA | 0.4821 | -0.6685 to 1.633 | No | ns | 0.9315 |
| NH vs. CA | 0.4662 | -0.9251 to 1.858 | No | ns | 0.982 |
| VT vs. RI | 6.573 | 0.5221 to 12.62 | Yes | * | 0.0215 |
| VT vs. MA | 7.024 | 1.21 to 12.84 | Yes | ** | 0.0056 |
| VT vs. NM | 5.398 | -0.4185 to 11.22 | No | ns | 0.0938 |
| VT vs. CO | 6.884 | 1.099 to 12.67 | Yes | ** | 0.007 |
| VT vs. WA | 7.021 | 1.253 to 12.79 | Yes | ** | 0.0051 |
| VT vs. CA | 7.005 | 1.185 to 12.83 | Yes | ** | 0.0059 |
| RI vs. MA | 0.451 | -1.549 to 2.451 | No | ns | 0.9988 |
| RI vs. NM | -1.175 | -3.183 to 0.8342 | No | ns | 0.6724 |
| RI vs. CO | 0.3115 | -1.605 to 2.228 | No | ns | 0.9999 |
| RI vs. WA | 0.4481 | -1.413 to 2.309 | No | ns | 0.9981 |
| RI vs. CA | 0.4322 | -1.587 to 2.451 | No | ns | 0.9992 |
| MA vs. NM | -1.626 | -2.731 to -0.5199 | Yes | *** | 0.0002 |
| MA vs. CO | -0.1394 | -1.067 to 0.7877 | No | ns | >0.9999 |
| MA vs. WA | -0.002863 | -0.8103 to 0.8046 | No | ns | >0.9999 |
| MA vs. CA | -0.0188 | -1.143 to 1.105 | No | ns | >0.9999 |
| NM vs. CO | 1.486 | 0.5394 to 2.433 | Yes | **** | <0.0001 |
| NM vs. WA | 1.623 | 0.7929 to 2.452 | Yes | **** | <0.0001 |
| NM vs. CA | 1.607 | 0.4664 to 2.747 | Yes | *** | 0.0004 |
| CO vs. WA | 0.1366 | -0.4342 to 0.7073 | No | ns | 0.9982 |
| CO vs. CA | 0.1206 | -0.8476 to 1.089 | No | ns | >0.9999 |
| WA vs. CA | -0.01593 | -0.8703 to 0.8384 | No | ns | >0.9999 |

ns = not statistically significant.
