# Supplementary material for: Mapping cannabis potency in medical and recreational programs in the United States
Source: PLoS One. 2020 Mar 26;15(3):e0230167. doi: 10.1371/journal.pone.0230167 (PMC7098613; doi:10.1371/journal.pone.0230167)
Supplement: S6 Table — ND = no data (DOCX) [file pone.0230167.s010.docx]

**S6 Table. Descriptive statistics for THC concentrations (%) in all products offered in each sampled state separated by % THC categories (<5%, >5<10% THC, >10<15% THC, >15% THC).**

| <5% THC |  |  |  |  |  |  |  |  |  |
| --- | --- | --- | --- | --- | --- | --- | --- | --- | --- |
|  | ME | NH | VT | RI | MA | NM | CO | WA | CA |
| 25% Percentile | ND | 0.35 | ND | 1.44 | 0.16 | 1 | 1 | 0.785 | 0.53 |
| Median | ND | 0.665 | ND | 1.44 | 0.9 | 4.12 | 2.4 | 1.1 | 0.7 |
| 75% Percentile | ND | 1.818 | ND | 1.44 | 3.7 | 4.35 | 4.05 | 3.32 | 3.65 |
| Mean | ND | 1.28 | ND | 1.44 | 1.702 | 3.255 | 2.342 | 1.922 | 1.62 |
| Std. Deviation | ND | 1.754 | ND | 0 | 1.687 | 1.545 | 1.45 | 1.415 | 1.843 |
| Std. Error of Mean | ND | 0.7161 | ND | 0 | 0.468 | 0.3372 | 0.3745 | 0.1756 | 0.6516 |
|  |  |  |  |  |  |  |  |  |  |
| >5<10% THC |  |  |  |  |  |  |  |  |  |
|  | ME | NH | VT | RI | MA | NM | CO | WA | CA |
| 25% Percentile | 6.64 | 7.95 | 6.21 | 7.908 | 5.5 | 6.53 | 5.588 | 5.9 | 5.72 |
| Median | 8.22 | 9.11 | 7.105 | 9.401 | 7 | 8 | 6.81 | 7.4 | 7.2 |
| 75% Percentile | 9.8 | 9.11 | 8 | 9.625 | 7.4 | 8.135 | 8.025 | 8.4 | 8.55 |
| Mean | 8.22 | 8.773 | 7.105 | 8.978 | 6.883 | 7.564 | 6.878 | 7.323 | 7.199 |
| Std. Deviation | 2.234 | 0.5623 | 1.033 | 1.055 | 1.258 | 1.223 | 1.507 | 1.463 | 1.611 |
| Std. Error of Mean | 1.58 | 0.2125 | 0.5167 | 0.5274 | 0.3247 | 0.2445 | 0.2325 | 0.1569 | 0.3908 |
|  |  |  |  |  |  |  |  |  |  |
| >10<15% THC |  |  |  |  |  |  |  |  |  |
|  | ME | NH | VT | RI | MA | NM | CO | WA | CA |
| 25% Percentile | 12.39 | 10.22 | 14 | ND | 11.5 | 12.51 | 12.08 | 12.3 | 12.3 |
| Median | 13.94 | 12.02 | 14 | ND | 13.7 | 14 | 13.93 | 13.72 | 13.8 |
| 75% Percentile | 14.04 | 13.85 | 14 | ND | 14.36 | 14.85 | 14.67 | 14.5 | 14.51 |
| Mean | 13.27 | 12.03 | 14 | ND | 12.98 | 13.49 | 13.33 | 13.36 | 13.42 |
| Std. Deviation | 1.028 | 2.046 | 0 | ND | 1.631 | 1.489 | 1.536 | 1.401 | 1.304 |
| Std. Error of Mean | 0.3427 | 1.023 | 0 | ND | 0.3199 | 0.1956 | 0.224 | 0.1125 | 0.1506 |
|  |  |  |  |  |  |  |  |  |  |
| >15% THC |  |  |  |  |  |  |  |  |  |
|  | ME | NH | VT | RI | MA | NM | CO | WA | CA |
| 25% Percentile | 17.4 | 19.69 | 17.34 | 20.12 | 18.88 | 18.05 | 19.66 | 19.5 | 17.67 |
| Median | 19.88 | 22.45 | 18 | 20.69 | 21.5 | 20.57 | 22.2 | 21.56 | 19.75 |
| 75% Percentile | 21.25 | 24.65 | 18 | 23.96 | 23.51 | 23.03 | 25.16 | 24 | 22.14 |
| Mean | 19.77 | 22.82 | 17.45 | 21.29 | 21.48 | 20.92 | 22.69 | 22.45 | 20.42 |
| Std. Deviation | 2.92 | 4.034 | 0.9655 | 2.985 | 3.364 | 3.783 | 4.153 | 5.058 | 4.293 |
| Std. Error of Mean | 0.5727 | 0.4276 | 0.2493 | 0.45 | 0.2018 | 0.1593 | 0.1226 | 0.07045 | 0.1908 |

ND= no data
