# Supplementary material for: Mapping cannabis potency in medical and recreational programs in the United States
Source: PLoS One. 2020 Mar 26;15(3):e0230167. doi: 10.1371/journal.pone.0230167 (PMC7098613; doi:10.1371/journal.pone.0230167)
Supplement: S7 Table — One-way ANOVA followed by Turkey’s multiple comparisons test was used, and P values are reported. A P<0.05 was considered statistically significant. ns = not statistically significant. (DOCX) [file pone.0230167.s011.docx]

**S7 Table. Comparisons of THC concentrations (%) in all products between each sampled state separated by % THC categories (<5%, >5<10% THC, >10<15% THC, >15% THC).** One-way ANOVA followed by Turkey’s multiple comparisons test was used, and P values are reported. A P<0.05 was considered statistically significant.

| Number of families | 1 |  |  |  |  |
| --- | --- | --- | --- | --- | --- |
| Number of comparisons per family | 36 |  |  |  |  |
| Alpha | 0.05 |  |  |  |  |
| Df | 7816 |  |  |  |  |
| Tukey's multiple comparisons test | Mean Diff. | 95.00% CI of diff. | Significant? | Summary | Adjusted P Value |
| ME vs. NH | -3.053 | -6.318 to 0.2126 | No | ns | 0.0886 |
| ME vs. VT | 2.321 | -2.428 to 7.07 | No | ns | 0.8483 |
| ME vs. RI | -1.517 | -5.14 to 2.107 | No | ns | 0.932 |
| ME vs. MA | -1.714 | -4.718 to 1.29 | No | ns | 0.7018 |
| ME vs. NM | -1.149 | -4.087 to 1.789 | No | ns | 0.9538 |
| ME vs. CO | -2.922 | -5.827 to -0.01717 | Yes | * | 0.0473 |
| ME vs. WA | -2.683 | -5.563 to 0.1972 | No | ns | 0.0912 |
| ME vs. CA | -0.6492 | -3.595 to 2.296 | No | ns | 0.999 |
| NH vs. VT | 5.374 | 1.285 to 9.462 | Yes | ** | 0.0015 |
| NH vs. RI | 1.536 | -1.163 to 4.235 | No | ns | 0.7052 |
| NH vs. MA | 1.338 | -0.4455 to 3.122 | No | ns | 0.3253 |
| NH vs. NM | 1.904 | 0.2331 to 3.574 | Yes | * | 0.0122 |
| NH vs. CO | 0.1306 | -1.481 to 1.742 | No | ns | >0.9999 |
| NH vs. WA | 0.3701 | -1.196 to 1.936 | No | ns | 0.9983 |
| NH vs. CA | 2.404 | 0.7199 to 4.087 | Yes | *** | 0.0003 |
| VT vs. RI | -3.838 | -8.217 to 0.5418 | No | ns | 0.1412 |
| VT vs. MA | -4.035 | -7.918 to -0.1525 | Yes | * | 0.0345 |
| VT vs. NM | -3.47 | -7.302 to 0.362 | No | ns | 0.1126 |
| VT vs. CO | -5.243 | -9.05 to -1.436 | Yes | *** | 0.0007 |
| VT vs. WA | -5.004 | -8.791 to -1.216 | Yes | ** | 0.0014 |
| VT vs. CA | -2.97 | -6.808 to 0.8675 | No | ns | 0.2832 |
| RI vs. MA | -0.1975 | -2.574 to 2.179 | No | ns | >0.9999 |
| RI vs. NM | 0.3677 | -1.925 to 2.66 | No | ns | >0.9999 |
| RI vs. CO | -1.405 | -3.656 to 0.8447 | No | ns | 0.5872 |
| RI vs. WA | -1.166 | -3.384 to 1.052 | No | ns | 0.7877 |
| RI vs. CA | 0.8675 | -1.435 to 3.17 | No | ns | 0.9629 |
| MA vs. NM | 0.5653 | -0.5081 to 1.639 | No | ns | 0.7862 |
| MA vs. CO | -1.208 | -2.187 to -0.2288 | Yes | ** | 0.0041 |
| MA vs. WA | -0.9684 | -1.87 to -0.06652 | Yes | * | 0.0245 |
| MA vs. CA | 1.065 | -0.02848 to 2.159 | No | ns | 0.0633 |
| NM vs. CO | -1.773 | -2.526 to -1.02 | Yes | **** | <0.0001 |
| NM vs. WA | -1.534 | -2.183 to -0.884 | Yes | **** | <0.0001 |
| NM vs. CA | 0.4998 | -0.3971 to 1.397 | No | ns | 0.7288 |
| CO vs. WA | 0.2395 | -0.2385 to 0.7175 | No | ns | 0.8293 |
| CO vs. CA | 2.273 | 1.491 to 3.055 | Yes | **** | <0.0001 |
| WA vs. CA | 2.033 | 1.351 to 2.716 | Yes | **** | <0.0001 |

ns = not statistically significant.
