# Supplementary material for: Mapping cannabis potency in medical and recreational programs in the United States
Source: PLoS One. 2020 Mar 26;15(3):e0230167. doi: 10.1371/journal.pone.0230167 (PMC7098613; doi:10.1371/journal.pone.0230167)
Supplement: S8 Table — ND = no data (DOCX) [file pone.0230167.s012.docx]

**S8 Table. Descriptive statistics for CBD concentrations (%) in all products offered in each sampled state separated by % THC categories (<5%, >5<10% THC, >10<15% THC, >15% THC).**

| CBD in <5% |  |  |  |  |  |  |  |  |  |
| --- | --- | --- | --- | --- | --- | --- | --- | --- | --- |
|  | ME | NH | VT | RI | MA | NM | CO | WA | CA |
| 25% Percentile | ND | 7.85 | ND | 12.71 | 0.05 | 8.698 | 12.18 | 8.325 | 11.76 |
| Median | ND | 13.75 | ND | 12.71 | 7.2 | 11.2 | 13.95 | 13 | 12.6 |
| 75% Percentile | ND | 14.47 | ND | 12.71 | 16.2 | 13.72 | 15.73 | 16.33 | 15.09 |
| Mean | ND | 12.04 | ND | 12.71 | 8.442 | 11.59 | 14.96 | 12.02 | 13.23 |
| Std. Deviation | ND | 3.264 | ND | 0 | 9.33 | 2.784 | 3.551 | 6.014 | 2.075 |
| Std. Error of Mean | ND | 1.333 | ND | 0 | 2.588 | 0.6224 | 1.025 | 0.7517 | 0.7843 |
|  |  |  |  |  |  |  |  |  |  |
| CBD in >5<10% |  |  |  |  |  |  |  |  |  |
|  | ME | NH | VT | RI | MA | NM | CO | WA | CA |
| 25% Percentile | 0.13 | 9.16 | 8 | 8.244 | 0.0025 | 10.69 | 7.85 | 8.85 | 7.475 |
| Median | 3.67 | 11.28 | 8.345 | 8.495 | 6.17 | 12 | 9.155 | 10.2 | 8.505 |
| 75% Percentile | 7.21 | 11.28 | 8.69 | 10.94 | 12.68 | 14 | 12.14 | 13.35 | 11.63 |
|  |  |  |  |  |  |  |  |  |  |
| Mean | 3.67 | 11.09 | 8.345 | 9.228 | 6.637 | 12.09 | 10.08 | 11.17 | 8.993 |
| Std. Deviation | 5.006 | 1.687 | 0.3984 | 1.696 | 6.258 | 3.854 | 2.861 | 4.984 | 4.316 |
| Std. Error of Mean | 3.54 | 0.6377 | 0.1992 | 0.8479 | 1.399 | 0.7707 | 0.4768 | 0.5537 | 1.365 |
|  |  |  |  |  |  |  |  |  |  |
| CBD in >10<15% |  |  |  |  |  |  |  |  |  |
|  | ME | NH | VT | RI | MA | NM | CO | WA | CA |
| 25% Percentile | 0.175 | 0.06 | ND | ND | 0 | 0 | 0 | 0.2 | 0.015 |
| Median | 0.29 | 6.625 | ND | ND | 0.1 | 0.29 | 0.02 | 0.3 | 0.03 |
| 75% Percentile | 0.3975 | 14.05 | ND | ND | 5.745 | 8.57 | 7.6 | 1.2 | 4.1 |
| Mean | 0.2875 | 6.913 | ND | ND | 2.182 | 3.527 | 3.44 | 2.517 | 2.345 |
| Std. Deviation | 0.117 | 7.916 | ND | ND | 3.431 | 4.804 | 5.768 | 4.178 | 4.364 |
| Std. Error of Mean | 0.05851 | 3.958 | ND | ND | 0.8321 | 1.048 | 1.259 | 0.5439 | 0.9524 |
|  |  |  |  |  |  |  |  |  |  |
| CBD in >15% |  |  |  |  |  |  |  |  |  |
|  | ME | NH | VT | RI | MA | NM | CO | WA | CA |
| 25% Percentile | 0.235 | 0.05 | ND | 0.01 | 0 | 0 | 0 | 0.06 | 0 |
| Median | 0.46 | 0.05 | ND | 0.744 | 0.1 | 0.01 | 0 | 0.2 | 0.04 |
| 75% Percentile | 0.915 | 0.06 | ND | 1.002 | 0.1 | 0.07 | 0.02 | 0.5 | 0.1075 |
| Mean | 0.6069 | 0.1383 | ND | 0.5733 | 0.09278 | 0.09462 | 0.2632 | 0.5656 | 0.2094 |
| Std. Deviation | 0.5155 | 0.7406 | ND | 0.5268 | 0.1783 | 0.1925 | 1.514 | 1.632 | 0.625 |
| Std. Error of Mean | 0.143 | 0.07894 | ND | 0.09035 | 0.01329 | 0.01599 | 0.07311 | 0.03611 | 0.04941 |

ND= no data
