# Supplementary material for: Mapping cannabis potency in medical and recreational programs in the United States
Source: PLoS One. 2020 Mar 26;15(3):e0230167. doi: 10.1371/journal.pone.0230167 (PMC7098613; doi:10.1371/journal.pone.0230167)
Supplement: S9 Table — (DOCX) [file pone.0230167.s013.docx]

**S9 Table. Descriptive statistics for THC (%, top) and CBD concentrations (%, bottom) in all products offered in CO and WA medical and recreational programs.**

| %THC | Medical | | Recreations | |
| --- | --- | --- | --- | --- |
|  | CO | WA | CO | WA |
| 25% Percentile | 18.45 | 19 | 19.06 | 19 |
| Median | 21.6 | 21.26 | 22 | 21.3 |
| 75% Percentile | 24.9 | 23.9 | 25 | 23.9 |
| Mean | 21.37 | 21.56 | 21.72 | 21.87 |
| Std. Deviation | 5.8 | 5.46 | 5.444 | 6.322 |
| Std. Error of Mean | 0.2485 | 0.1026 | 0.2047 | 0.1233 |
|  |  |  |  |  |
| %CBD |  |  |  |  |
|  | CO | WA | CO | WA |
| 25% Percentile | 0 | 0 | 0 | 0.2 |
| Median | 0 | 0.19 | 0 | 0.3 |
| 75% Percentile | 0.56 | 0.4 | 0.26 | 0.9 |
| Mean | 1.718 | 1.15 | 1.269 | 1.503 |
| Std. Deviation | 4.291 | 3.468 | 3.61 | 3.476 |
| Std. Error of Mean | 0.294 | 0.1026 | 0.2138 | 0.1045 |
