# Supplementary material for: Mapping cannabis potency in medical and recreational programs in the United States
Source: PLoS One. 2020 Mar 26;15(3):e0230167. doi: 10.1371/journal.pone.0230167 (PMC7098613; doi:10.1371/journal.pone.0230167)
Supplement: S10 Table — (DOCX) [file pone.0230167.s014.docx]

**S10 Table. Descriptive statistics for THC concentrations (%) in all products offered in CO and WA medical and recreational programs separated by % THC categories (<5%, >5<10% THC, >10<15% THC, >15% THC).**

| <5% THC | Medical | | Recreational | |
| --- | --- | --- | --- | --- |
|  | CO | WA | CO | WA |
| 25% Percentile | 1.2 | 0.7 | 0.8925 | 0.9 |
| Median | 2.63 | 1.2 | 2 | 1.1 |
| 75% Percentile | 4.063 | 3.57 | 3.48 | 1.65 |
| Mean | 2.535 | 2.12 | 2.214 | 1.624 |
| Std. Deviation | 1.479 | 1.481 | 1.506 | 1.282 |
| Std. Error of Mean | 0.6037 | 0.2371 | 0.502 | 0.2514 |
|  |  |  |  |  |
| >5<10% THC | Medical | | Recreational | |
|  | CO | WA | CO | WA |
| 25% Percentile | 5.728 | 5.9 | 5.02 | 5.925 |
| Median | 6.56 | 6.7 | 6.9 | 7.7 |
| 75% Percentile | 8.73 | 8.1 | 7.925 | 8.825 |
| Mean | 6.987 | 7.108 | 6.758 | 7.467 |
| Std. Deviation | 1.51 | 1.418 | 1.533 | 1.489 |
| Std. Error of Mean | 0.322 | 0.2396 | 0.3428 | 0.2065 |
|  |  |  |  |  |
| >10<15% | Medical | | Recreational | |
|  | CO | WA | CO | WA |
| 25% Percentile | 12.14 | 12.66 | 12.26 | 12.2 |
| Median | 14 | 14 | 14 | 13.6 |
| 75% Percentile | 14.67 | 14.6 | 14.67 | 14.5 |
| Mean | 13.4 | 13.57 | 13.38 | 13.26 |
| Std. Deviation | 1.502 | 1.274 | 1.593 | 1.453 |
| Std. Error of Mean | 0.1989 | 0.1249 | 0.2488 | 0.1432 |
|  |  |  |  |  |
| >15% THC | Medical | | Recreational | |
|  | CO | WA | CO | WA |
| 25% Percentile | 19.38 | 19.4 | 20 | 19.6 |
| Median | 22.12 | 21.5 | 22.27 | 21.6 |
| 75% Percentile | 25.43 | 24 | 25.1 | 24.1 |
| Mean | 22.69 | 22.18 | 22.69 | 22.76 |
| Std. Deviation | 4.267 | 4.621 | 4.07 | 5.487 |
| Std. Error of Mean | 0.1928 | 0.08882 | 0.1587 | 0.1109 |
