# Supplementary material for: Mapping cannabis potency in medical and recreational programs in the United States
Source: PLoS One. 2020 Mar 26;15(3):e0230167. doi: 10.1371/journal.pone.0230167 (PMC7098613; doi:10.1371/journal.pone.0230167)
Supplement: S11 Table — (DOCX) [file pone.0230167.s015.docx]

**S11 Table. Descriptive statistics for CBD concentrations (%) in all products offered in CO and WA medical and recreational programs separated by % THC categories (<5%, >5<10% THC, >10<15% THC, >15% THC).**

| CBD in <5% THC | Medical | | Recreational | | |
| --- | --- | --- | --- | --- | --- |
|  | CO | WA | CO | WA | |
| 25% Percentile | 12 | 8.18 | 13.25 | 8.15 | |
| Median | 12.18 | 12.39 | 14.51 | 13 | |
| 75% Percentile | 18.97 | 16.6 | 15.1 | 15.98 | |
| Mean | 14.82 | 12.38 | 15.06 | 11.49 | |
| Std. Deviation | 4.35 | 5.684 | 3.237 | 6.545 | |
| Std. Error of Mean | 1.945 | 0.9221 | 1.223 | 1.284 | |
|  |  |  |  |  | |
| CBD in >5<10% THC | Medical | | Recreational | | |
|  | CO | WA | CO | | WA |
| 25% Percentile | 8 | 8.55 | 7.8 | | 9.3 |
| Median | 9.11 | 11.2 | 9.8 | | 9.8 |
| 75% Percentile | 12.14 | 14 | 12.27 | | 12.9 |
| Mean | 10.05 | 11.34 | 10.1 | | 11.08 |
| Std. Deviation | 2.915 | 6.052 | 2.889 | | 4.298 |
| Std. Error of Mean | 0.6687 | 1.105 | 0.7006 | | 0.6018 |
|  |  |  |  | |  |
| CBD in >10<15% THC | Medical | | Recreational | | |
|  | CO | WA | CO | | WA |
| 25% Percentile | 0 | 0.1 | 0 | | 0.2 |
| Median | 0.02 | 0.3 | 0.02 | | 0.4 |
| 75% Percentile | 7.6 | 0.575 | 9.015 | | 8 |
| Mean | 3.273 | 1.135 | 3.711 | | 3.464 |
| Std. Deviation | 5.174 | 2.695 | 7.002 | | 4.753 |
| Std. Error of Mean | 1.435 | 0.55 | 2.476 | | 0.8034 |
|  |  |  |  | |  |
| CBD in >15% THC | Medical | | Recreational | | |
|  | CO | WA | CO | | WA |
| 25% Percentile | 0 | 0 | 0 | | 0.2 |
| Median | 0 | 0.13 | 0 | | 0.3 |
| 75% Percentile | 0 | 0.39 | 0.04 | | 0.7 |
| Mean | 0.3309 | 0.4536 | 0.2161 | | 0.6839 |
| Std. Deviation | 2.145 | 1.657 | 0.8333 | | 1.597 |
| Std. Error of Mean | 0.1617 | 0.05115 | 0.05239 | | 0.05068 |
