# Supplementary material for: Mapping cannabis potency in medical and recreational programs in the United States
Source: PLoS One. 2020 Mar 26;15(3):e0230167. doi: 10.1371/journal.pone.0230167 (PMC7098613; doi:10.1371/journal.pone.0230167)
Supplement: Attachment — Submitted filename: PONE-D-19-25611_reviewer.pdf [file pone.0230167.s016.pdf]

PLOS ONE

Mapping cannabis potency in medical and recreational programs in the United States.

--Manuscript Draft--

Manuscript Number:	PONE-D-19-25611
Article Type:	Research Article
Full Title:	Mapping cannabis potency in medical and recreational programs in the United States.
Short Title:	Cannabis potency in dispensaries of the U.S.A.
Corresponding Author:	E. Alfonso Romero-Sandoval<br>Wake Forest University School of Medicine<br>Winston Salem, NC UNITED STATES
Keywords:	Pain; Dispensaries; marijuana; THC; CBD; tetrahydrocannabinol; cannabidiol
Abstract:	Cannabis related online searches are associated with positive attitudes toward medical cannabis, particularly when information is obtained from dispensaries. Since pain is the main reason for medicinal cannabis use, information from dispensary websites has the potential to shape the attitude of pain patients towards cannabis. This is relevant because cannabis has demonstrated efficacy in neuropathic pain with low tetrahydrocannabinol (THC) concentrations (< 5-10%), in contrast to potent cannabis (>15% THC), which is highly rewarded in the recreational realm. The role of CBD in pain is not clear, however it has gained popularity. Thus, we hypothesize that the potency of medical cannabis that is advertised online is similar to the cannabis advertised for recreational purposes, which would potentially create a misconception towards medical cannabis. The current lack of knowledge surrounding advertised potencies in the legal cannabis market limits the ability to generate clear policies regarding online advertising to protect patients that are willing to use cannabis for their condition. Thus, we evaluated the advertised THC and CBD content of cannabis products offered online in dispensaries in the United States to determine products' suitability to medicinal use and compare the strength of strains offered in legal medical and recreational programs. We recorded THC and CBD concentrations for all herb cannabis products provided by dispensary websites and compared them between or within states. Four Western states (CA, CO, NM, WA) and five Northeastern states (ME, MA, NH, RI, VT) were included. A total of 8,505 cannabis strains across 653 dispensaries were sampled. Despite the clear differences between medicinal and recreational uses of cannabis, the average THC concentration advertised online in medicinal programs was similar (19.2% ±6.2) to recreational programs (21.5% ±6.0) when compared between states with different programs, or between medicinal and recreational programs within the same states (CO or WA). Lower CBD concentrations accompanied higher THC products. The majority of products, regardless of medicinal or recreational programs, were advertised to have >15% THC (70.3% - 91.4% of products). These stated concentrations seem unsuitable for medicinal purposes, particularly for patients with chronic neuropathic pain. Therefore, this information could induce the misconception that high potency cannabis is safe to treat pain. This data is consistent with reports in which THC and CBD in products from legal dispensaries or in nationwide products from the illegal market were actually measured, which indicates that patients consuming these products may be at risk of acute intoxication or long-term side effects. Our study offers grounds to develop policies that help prevent misconceptions toward cannabis and reduce risks in pain patients.
Order of Authors:	Mary Catherine Cash
	Katharine Cunnane
	Chuyin Fan
	E. Alfonso Romero-Sandoval
Additional Information:	
Question	Response
Financial Disclosure	Funding from Department of Anesthesiology at Wake Forest University School of

Powered by Editorial Manager® and ProduXion Manager® from Aries Systems Corporation

Enter a financial disclosure statement that describes the sources of funding for the work included in this submission. Review the submission guidelines for detailed requirements. View published research articles from *PLOS ONE* for specific examples.<br><br>This statement is required for submission and **will appear in the published article** if the submission is accepted. Please make sure it is accurate.<br><br>**Unfunded studies**<br>Enter: *The author(s) received no specific funding for this work.*<br><br>**Funded studies**<br>Enter a statement with the following details:<br>• Initials of the authors who received each award<br>• Grant numbers awarded to each author<br>• The full name of each funder<br>• URL of each funder website<br>• Did the sponsors or funders play any role in the study design, data collection and analysis, decision to publish, or preparation of the manuscript?<br>• **NO** - Include this sentence at the end of your statement: *The funders had no role in study design, data collection and analysis, decision to publish, or preparation of the manuscript.*<br>• **YES** - Specify the role(s) played.<br><br>* typeset	Medicine (E.A.R.s.).<br>The funders had no role in study design, data collection and analysis, decision to publish, or preparation of the manuscript.
**Competing Interests**<br><br>Use the instructions below to enter a competing interest statement for this submission. On behalf of all authors, disclose any competing interests that could be perceived to bias this work—acknowledging all financial support and any other relevant financial or non-financial competing interests.<br><br>This statement **will appear in the published article** if the submission is accepted. Please make sure it is accurate. View published research articles	The authors have declared that no competing interests exist.

Powered by Editorial Manager® and ProduXion Manager® from Aries Systems Corporation

from *PLOS ONE* for specific examples.<br><br>**NO authors have competing interests**<br><br>Enter: *The authors have declared that no competing interests exist.*<br><br>**Authors with competing interests**<br><br>Enter competing interest details beginning with this statement:<br><br>*I have read the journal's policy and the authors of this manuscript have the following competing interests: [insert competing interests here]*<br><br>* typeset	
**Ethics Statement**<br><br>Enter an ethics statement for this submission. This statement is required if the study involved:<br><br>• Human participants<br>• Human specimens or tissue<br>• Vertebrate animals or cephalopods<br>• Vertebrate embryos or tissues<br>• Field research<br><br>Write "N/A" if the submission does not require an ethics statement.<br><br>General guidance is provided below. Consult the submission guidelines for detailed instructions. **Make sure that all information entered here is included in the Methods section of the manuscript.**	N/A

**Format for specific study types**<br><br>**Human Subject Research (involving human participants and/or tissue)**<br>• Give the name of the institutional review board or ethics committee that approved the study<br>• Include the approval number and/or a statement indicating approval of this research<br>• Indicate the form of consent obtained (written/oral) or the reason that consent was not obtained (e.g. the data were analyzed anonymously)<br><br>**Animal Research (involving vertebrate animals, embryos or tissues)**<br>• Provide the name of the Institutional Animal Care and Use Committee (IACUC) or other relevant ethics board that reviewed the study protocol, and indicate whether they approved this research or granted a formal waiver of ethical approval<br>• Include an approval number if one was obtained<br>• If the study involved *non-human primates*, add *additional details* about animal welfare and steps taken to ameliorate suffering<br>• If anesthesia, euthanasia, or any kind of animal sacrifice is part of the study, include briefly which substances and/or methods were applied<br><br>**Field Research**<br><br>Include the following details if this study involves the collection of plant, animal, or other materials from a natural setting:<br>• Field permit number<br>• Name of the institution or relevant body that granted permission	
**Data Availability**<br><br>Authors are required to make all data underlying the findings described fully available, without restriction, and from the time of publication. PLOS allows rare exceptions to address legal and ethical concerns. See the PLOS Data Policy and FAQ for detailed information.	Yes - all data are fully available without restriction

A Data Availability Statement describing where the data can be found is required at submission. Your answers to this question constitute the Data Availability Statement and **will be published in the article**, if accepted.<br><br>**Important:** Stating 'data available on request from the author' is not sufficient. If your data are only available upon request, select 'No' for the first question and explain your exceptional situation in the text box.<br><br>Do the authors confirm that all data underlying the findings described in their manuscript are fully available without restriction?	
**Describe where the data may be found in full sentences. If you are copying our sample text, replace any instances of XXX with the appropriate details.**<br><br>• If the data are **held or will be held in a public repository**, include URLs, accession numbers or DOIs. If this information will only be available after acceptance, indicate this by ticking the box below. For example: *All XXX files are available from the XXX database (accession number(s) XXX, XXX.).*<br>• If the data are all contained **within the manuscript and/or Supporting Information files**, enter the following: *All relevant data are within the manuscript and its Supporting Information files.*<br>• If neither of these applies but you are able to provide **details of access elsewhere**, with or without limitations, please do so. For example:<br><br>*Data cannot be shared publicly because of [XXX]. Data are available from the XXX Institutional Data Access / Ethics Committee (contact via XXX) for researchers who meet the criteria for access to confidential data.*<br><br>*The data underlying the results presented in the study are available from (include the name of the third party*	All relevant data are within the manuscript and its Supporting Information files

and contact information or URL).<br>• This text is appropriate if the data are owned by a third party and authors do not have permission to share the data.<br><br>* typeset	
Additional data availability information:	

Manuscript

Click here to download Manuscript Mapping cannabis potency USA - PloS One.docx

Mapping cannabis potency in medical and recreational programs in the United States.

Mary Catherine Cash, Pharm.D. Candidate[1*]; Katharine Cunnane, BS Candidate[2*]; Chuyin Fan, Pharm.D. Candidate[1]; E. Alfonso Romero-Sandoval, M.D., Ph.D.[2]

*These authors contributed equally to this article.

[1]*The University of North Carolina Eshelman School of Pharmacy, Chapel Hill, NC*
[2]*Wake Forest University School of Medicine, Department of Anesthesiology, Winston-Salem, NC*

Authors emails:

Mary Catherine Cash, mccash@email.unc.edu

Katharine Cunnane, cunnka16@wfu.edu

Chuyin Fan, chuyinf@email.unc.edu

E. Alfonso Romero-Sandoval, earomero.sandoval@gmail.com

***Corresponding author**: E. Alfonso Romero-Sandoval, M.D., Ph.D.

E-mail: earomero.sandoval@gmail.com

Address: Wake Forest School of Medicine, 1 Medical Center Blvd, Winston-Salem, NC 27157, USA.

Phone: 336-716-2725

Working Title: Cannabis potency in dispensaries of the U.S.A

Figures: 9

Tables: 2

Supplemental figure: 1

Supplemental tables: 2

Kew words: Dispensaries; marijuana; THC; CBD; tetrahydrocannabinol; cannabidiol.

Abstract. Cannabis related online searches are associated with positive attitudes toward medical cannabis, particularly when information is obtained from dispensaries. Since pain is the main reason for medicinal cannabis use, information from dispensary websites has the potential to shape the attitude of pain patients towards cannabis. This is relevant because cannabis has demonstrated efficacy in neuropathic pain with low tetrahydrocannabinol (THC) concentrations (< 5-10%), in contrast to potent cannabis (>15% THC), which is highly rewarded in the recreational realm. The role of CBD in pain is not clear, however it has gained popularity. Thus, we hypothesize that the potency of medical cannabis that is advertised online is similar to the cannabis advertised for recreational purposes, which would potentially create a misconception towards medical cannabis. The current lack of knowledge surrounding advertised potencies in the legal cannabis market limits the ability to generate clear policies regarding online advertising to protect patients that are willing to use cannabis for their condition. Thus, we evaluated the advertised THC and CBD content of cannabis products offered online in dispensaries in the United States to determine products' suitability to medicinal use and compare the strength of strains offered in legal medical and recreational programs. We recorded THC and CBD concentrations for all herb cannabis products provided by dispensary websites and compared them between or within states. Four Western states (CA, CO, NM, WA) and five Northeastern states (ME, MA, NH, RI, VT) were included. A total of 8,505 cannabis strains across 653 dispensaries were sampled. Despite the clear differences between medicinal and recreational uses of cannabis, the average THC concentration advertised online in medicinal programs was similar (19.2% ±6.2) to recreational programs (21.5% ±6.0) when compared between states with different programs, or between medicinal and recreational programs within the same states (CO or WA). Lower CBD concentrations accompanied higher THC products. The majority of products, regardless of medicinal or recreational programs, were advertised to have >15% THC (70.3% - 91.4% of products). These stated concentrations seem unsuitable for medicinal purposes, particularly for patients with chronic neuropathic pain. Therefore, this information could induce the misconception that high potency cannabis is safe to treat pain. This data is consistent with reports in which THC and CBD in products from legal dispensaries or in nationwide products from the illegal market were actually measured, which indicates that

patients consuming these products may be at risk of acute intoxication or long-term side effects. Our study offers grounds to develop policies that help prevent misconceptions toward cannabis and reduce risks in pain patients.

Introduction

The practice of pain management has come under scrutiny in recent years with the rise of the opioid epidemic in the United States (U.S.). Physicians continue to search for alternatives when opioids, anticonvulsants, or antidepressants provide no relief or result in adverse effects. Cannabis offers an alternative to pain management, and states with legalized medical cannabis programs have witnessed a decline in the number of opioid prescriptions (1–3). Pain is the foremost reason patients visit cannabis dispensaries across the U.S. (1,4). As of April 2018, 29 states and Washington D.C. have legalized cannabis for medical use in the U.S.. Of those, 9 states and Washington D.C. have also legalized cannabis for recreational use. Twenty-seven states list pain as a qualifying condition. Thus, the U.S. represents a largely populated geographical area in which cannabis is becoming legal and accessible in a non-uniformly regulated market in contrast to other countries. This tendency towards cannabis legalization in the U.S. has been accompanied with robust dissemination of information using new technologies, namely online advertisements. In fact, marijuana or cannabis online searches have grown exponentially during the last decade across the U.S. (5). Interestingly, online presence of cannabis products is associated with positive attitudes towards the medicinal properties of cannabis (6). More importantly, it has been demonstrated that information provided by dispensaries is highly regarded by patients as safe and reliable (7). Providing wrong information via online advertisements represents a high risk for public health, as evidenced by the recent concern expressed by the Federal Drug Administration (FDA) towards online offers and claims about cannabis products (8). Therefore, understanding what information is provided online by dispensaries will provide a better understanding on how this shapes the attitude of pain patients towards cannabis products for medical purposes.

Inhaled cannabis has been proven effective for the treatment of various types of chronic pain ranging from neuropathic pain to diabetic nephropathy and has a more favorable pharmacokinetic profile than oral formulations (9–13). Thus, knowing what type of cannabis herbal products are offered online is relevant for pain patients. The main components found in cannabis are tetrahydrocannabinol (THC), the psychoactive constituent of cannabis, and cannabidiol (CBD), which is devoid of intoxicating effects. THC is the primary constituent of cannabis and is

responsible for its analgesic and euphoric effects, as well as its adverse effects. With an increase in THC content (%THC for inhaled formulations) analgesic effects are lost and adverse events increase (9–11). An array of studies have demonstrated efficacy in pain reduction with minimal and tolerable psychotropic effects with THC concentrations lower than 5% or 10% (9–11). Alternatively, CBD ameliorates the euphoric effects and counteracts the unintended adverse effects of THC (14). Thus, the ratio of THC to CBD seems to play a crucial role in the interaction between the two cannabinoids, contributing to the overall euphoric and therapeutic effects patients experience. Even though a recent article has described some aspects of U.S. dispensaries' online practices and information about cannabis (15), thus far there are no reports on the strength of cannabis products offered in legal medical cannabis programs across the United States (U.S.), and how this compares to recreational programs. The clinical relevance of such information relies on the fact that this information could be considered safe by patients, which may strongly influence their attitude towards cannabis as medicine (7). In addition, this study seeks to uncover whether the reported potency of cannabis products marketed online are in line with the few reports on the potency of products measured at different times, settings, techniques, and laboratories (16,17). This type of information is relevant from a policy or regulatory standpoint as it would allow us to evaluate the accuracy of advertised cannabis potency. Altogether, this information could guide more immediate decisions by regulatory agencies to request changes in online product offers from dispensaries just as the FDA has done recently through warning letters directed to remove online unsubstantiated claims of cannabis products (8).

Due to the continuous historic increase in THC content in cannabis products on the illegal market in the U.S. (18–20), we hypothesize that the THC content of cannabis offered in legal medical programs are higher than the ideal concentration required for the treatment of pain (<5-10%) and that the THC and CBD content of strains found in medicinal dispensaries are comparable to those found in recreational programs. This study aims to map the THC and CBD content available on dispensary websites in states with legalized medical and/or recreational cannabis programs in the Northeastern and Western regions of the U.S. in order to characterize the variety of products available to patients across the country. This study will also evaluate the appropriateness of available strains for medicinal use and determine whether a difference exists between the strength of cannabis offered by medical and

recreational programs. Our study has the potential to impact public health as it provides the foundation to develop and implement evidence-based policies and regulations for online herbal cannabis advertisement for medical purposes, which in turn will result in more realistic patient attitude towards cannabis.

Materials and Methods

Inclusion and Exclusion Criteria

States with legalized medical and/or recreational cannabis programs were identified. The number of licensed dispensaries in each state was determined. Dispensaries were evaluated for online presence. Those with established websites (not including a profile on Leafly or WeedMaps) were assessed for the availability of THC and CBD data. Only states that have legalized cannabis for the treatment of pain management were included. States or programs who do not allow for the inhaled administration of cannabis were excluded, as were those recreational cannabis programs that have yet to take effect despite being legalized (as was the case with Massachusetts and Maine). States were considered to have an "active" program if there were licensed dispensaries open and operating. Two distinct geographical locations containing a group of states meeting the above criteria were identified - the Northeast region and the Western region of the United States. Selected states from the Northeast region include Maine (ME), Massachusetts (MA), New Hampshire (NH), Rhode Island (RI), and Vermont (VT). Selected states from the Western region include Colorado (CO), New Mexico (NM), Washington (WA), and California (CA). Of the selected states, all Northeastern states and NM had legalized cannabis only for medicinal use. CO, WA and CA have legalized cannabis for recreational and medicinal use. Due to the variation in geographical size and cannabis program size between the Northeast and the West, each region had unique set of inclusion and exclusion criteria and a unique protocol for dispensary sampling. More information on licensed dispensaries, dispensaries sampled, and their inclusion criteria could be found in Supporting Information (Tables S1 and S2).

Northeast

States were considered for inclusion if more than 50% of its dispensaries had an online presence. In addition, states were only included if more than 50% of dispensaries provided THC content on their websites. A dispensary was deemed to have THC content available online if more than 50% of the strains available at that dispensary had THC content (%) listed on their website. The size of the medical and recreational cannabis programs in the Northeast made it possible to sample every licensed dispensary in each state for online presence and availability of THC content. Data for the Northeastern states was collected between March and May of 2018.

West

Due to the size of the medical and recreational cannabis programs in the West a representative portion of dispensaries was sampled in states with more than 100 dispensaries. The process by which dispensaries were selected varied from state to state depending on the availability of licensed dispensary data on government websites. Sampled dispensaries were geographically dispersed throughout each sampled state in order to maintain a representative sample. If a state had more than 100 licensed dispensaries, at least 30% were sampled for online presence and THC content. Western states were considered for inclusion if more than 50% of the sampled dispensaries had an online presence. The criteria for Northeastern states to have more than 50% of the dispensaries include THC content online was not considered when determining the inclusion of Western states as many Western dispensaries elect not to provide cannabinoid content on their websites. Similar to the Northeastern states, dispensaries were determined to have available THC content if more than 50% of strains listed on a dispensary website reported THC content. Specifics regarding the selection of dispensaries in the Western states may be found in the Supporting Information (Figure S1). Data for the Western states was collected between June and August of 2018.

Data Collection

Cannabinoid data was collected for all strains of Sativa, Indica, Hybrid, and CBD-rich flowers and pre-rolls. For strains that provided a range of THC or CBD, an average was calculated (i.e. THC 15-17%, a THC of 16% was

recorded). For THC or CBD reported as being less than or equal to a particular value, that number was reported as the THC or CBD value (i.e. CBD <0.05%, a CBD of 0.05% was recorded).

Statistical Analysis

Mean and standard deviation were determined for each state. Histograms containing THC and CBD concentrations were constructed and three concentration ranges were identified: THC <5%, THC 5-10%, and THC 10 -15%, and THC > 15%. Student's T test or One-way ANOVA and Turkey's multiple comparison test were used. A $p<0.05$ was considered statistically significant.

Results

We first quantified the THC and CBD content of all herb cannabis products offered in the surveyed dispensaries from states with legal medicinal marijuana programs and compared it with similar products offered in dispensaries from states with legal recreational/medicinal marijuana programs. All data per product and studied states are available in Supporting Information. The average THC concentration in products from medicinal programs was significantly lower (19.2% ± 6.2) than those found in products from recreational programs (21.5 % ± 6.0, $P<0.0001$, Figure 1A). The average CBD concentration in products from medicinal programs was also significantly higher (2.0% ± 4.5) than those found in products from recreational programs (1.5 % ± 4.2, $P<0.003$, Figure 1B). However, these average values are not representative of the wide range in the concentrations of THC and CBD. For example, products ranged from 0 to 35% THC in medical programs, while THC concentrations ranged from 0 to 45% in recreational programs. Most products in medical programs contain less than 5% CBD with some containing 15%, while most products in recreational programs contain between 0 to 18% CBD, with some above 20% and a few products above 40%[illegible]

We then sought to determine whether this variability persists in a state-by-state basis. To this end, we performed a similar quantification of the THC and CBD content of all herb cannabis products by state and conducted

comparisons between all states with legal medicinal and recreational/medicinal marijuana programs. We found that average THC concentrations were numerically similar in all surveyed states, but some statistical differences were found (ranging from 15.2% THC in VT to 21.72% THC in WA, Figure 2). Comparisons between states may be found in Table 1. When CBD was analyzed, we found that average CBD concentrations were more variable between states (ranging from 0.9% CBD in ME to 8.3% CBD in VT). We observed that the average CBD concentration in VT (8.3% CBD ± 0.4) was significantly higher when compared to ME (0.9% CBD ± 1.6, P= 0.03), MA (1.3% CBD ± 4.0, P = 0.03) and WA (1.3% CBD ± 3.5, P= 0.03). Additionally, we observed that the average CBD concentration in NM (2.9% CBD ± 5.2) was significantly higher when compared to MA (P= 0.002) and WA (P= <0.0001).

The large variability in THC and CBD concentration remains when the analysis was conducted in each studied state, which could result in a misleading hypothesis evaluation. Therefore, we conducted a more detailed analysis based on THC concentrations that represent the clinical or recreational adequacy of the products available in medicinal and/or recreational programs in the studied states. Thus, we divided herb products into 4 categories based on the level of THC: <5% THC, ≥5≤10% THC, >10≤15% THC and >15%THC. We observed in all states that the majority of THC products had >15% THC (ranging from 70.3% of products in ME to 91.4% of products in CO). Excluding VT and NH, the second most abundant category in all states was >10≤15% THC. In all states but VT and NH, the third most abundant category was ≥5≤10% THC. The least abundant category in all states except for NH was <5% THC. In VT, the second most abundant category was ≥5≤10% THC followed by >10≤15% THC. In New Hampshire, the second most abundant category was ≥5≤10% THC closely followed by the <5% THC category, with only 3.77% of products falling in the >10≤15% THC range. Percentage of products in each THC category may be found in Figure 3.

To further compare the potential variability in product THC concentrations in states with legal medicinal and recreational/medicinal marijuana programs, we plotted the concentration of THC in individual herb products in

each THC category separated by state (Figure 4). The most salient findings are that ME and VT offered no products in the <5% THC category, and RI only offered 1. These are states with only medicinal programs. ~~All states ranged their THC levels similarly in the other THC categories~~. However, in the >15% THC category, CO (ranging from 15.1% THC to 55% THC), WA (ranging from 15.1% THC to 90% THC), and CA (ranging from 15.01% THC to 88.73% THC) offered cannabis products that spanned a much wider range of THC concentrations than the other surveyed states. Significant difference among states in the >15% THC category could be found in Table 2.

In order to analyze the ratio of THC to CBD, the concentration of CBD in each product was separated by state and plotted within its THC category (Figure 5). In the <5% THC and ≥5≤10% THC categories, there were no significant differences in average CBD concentration between states, which were approximately 10% CBD. Interestingly, most of the Northeastern states (medical programs) have the smallest range of CBD content in these categories. However, WA and MA offered products with a much wider range of CBD concentrations (approximately ranging from 0% to 25% CBD) in the <5% THC category. It is noteworthy that MA, NM, CO, WA, and CA offered a variety of different CBD concentrations between 0% CBD and 30% CBD in the ≥5≤10% THC. In the >10≤15% and >10% THC categories, the levels of CBD were much lower than in the other THC categories. For example, the average concentration of CBD in products offered by CO in the >10≤15% THC category (11.5% CBD ± 19.6) was significantly higher when compared to MA (2.1% CBD ± 3.4, P= 0.04) and WA (2.5% CBD ± 4.2, P=0.002), whereas the rest of states have products with less than 3% CBD. In the >15% THC category, CBD concentrations were in average below 2% (NH did not offer any products with CBD in this THC category). We observed that the average CBD concentration in products from WA (0.6% CBD ± 1.6) was significantly higher when compared to NM (0.09% CBD ± 0.19, P= 0.01), MA (0.09% CBD ± 0.2, P= 0.003), and CO (0.26% CBD ± 1.5, P= 0.008).

Next, we selected the two states where medical and recreational cannabis products are sold separately. In CO, the majority of dispensaries offered separate medical and recreational menus within the same establishment. In Washington, most medical dispensaries existed as entirely separate entities from recreational dispensaries. Thus, we

were able to draw comparisons between recreational and medical strains of cannabis within the same state in two different scenarios.

The average THC concentrations were numerically similar in all CO and WA programs (approximately 21%, Figure 6). Both medical and recreational dispensaries in WA offered a wider variety of products, with some products containing well above 40% THC. CBD average concentrations were more variable in both states. We observed that the average CBD concentration in CO medical products (2.4% THC ± 7.5) was significantly higher than the average CBD concentrations of WA medical products (1.2% THC ± 3.5, P= 0.0001), CO recreational products (1.5% THC ± 4.8, P= 0.04), and WA recreational products (1.5% ± 3.5, P= 0.01).

Next, we divided herb products into the same 4 %THC categories as before (Figure 7). We observed in both medicinal and recreational programs, the majority of THC products had >15% THC (ranging from 89.4% of CO medical products to 95.7% of WA medical products). The second most abundant THC category in all conditions was >10≤15% THC. The third most abundant category in all conditions excluding WA medical was ≥5≤10% THC. In all states except for WA medical, the least abundant category was <5% THC. In WA medical products, the third most abundant category was <5% THC, making ≥5≤10% THC the least abundant category.

To further compare the distribution of THC concentrations of products in states with separate legal medicinal and recreational programs, the concentration of THC in individual cannabis products was plotted in each THC category, separated by state and program. We observed similar averages and ranges of THC concentrations in all conditions in <5%, ≥5≤10%, and >10≤15% THC categories. In the >15% THC category, WA shows a wider range of THC concentrations, but recreational products (22.76% THC ± 5.5) were significantly higher in THC than WA medicinal products (22.2% THC ± 2.2, P= 0.0004; Figure 8).

The concentration of CBD in each individual herb product was again separated by program and plotted within its THC category (Figure 9). Average CBD values were similar in all conditions in <5% and ≥5≤10% THC categories (11.5-15% in <5% THC category, 10.1-11.3 % in ≥5≤10% THC category), but WA displayed a wider range of CBD concentrations. In the >10≤15% THC category, average CBD concentrations varied greatly (ranging from 1.1% CBD in WA medical to 12.8% CBD in WA recreational), and average CBD concentration of CO medicinal products (12.8% CBD ± 21.0) was significantly higher when compared to WA recreational products (3.5% CBD ± 4.8, P= 0.04) and WA medicinal products (1.1% CBD ± 2.7, P= 0.01). In the >15% THC category, average CBD concentrations were numerically similar and low (<0.7%). However, there were a number of products with elevated CBD as well as elevated THC. CBD concentrations got as high as 27-30% in CO medical, WA medical, and WA recreational products. Products in CO recreational products only got as high as 10-12% CBD.

Discussion

The first major observation of our study was that the average concentration of THC in all states was two to three times the THC content known to be efficacious in the treatment of pain (i.e. >5-10%). The second major finding of our study was that a vast majority of strains in all states, including medical-only programs, contained THC designed for recreational use (i.e. > 15%). Patients who find this information in their online searches may subsequently deem high potency products suitable for medical purposes, placing themselves at higher risk of cannabis intoxication. Severe intoxication, hyperemesis, psychiatric symptoms, and severe cardiovascular events have been reported to be a major cause of cannabis-related visits to emergency departments in Colorado (21). Such undesirable adverse effects may lead to a perception of treatment failure in patients who have already failed traditional pain management therapies, while dependence may potentiate the long-term use of high potency cannabis. The prolonged use of high potency cannabis increases the risk for psychotic disorders by 5-fold in daily users compared to never users (22), increases the risk of memory impairment and paranoia (23–25), and is associated with cannabis admissions to drug treatment (26). People who use cannabis for medicinal purposes have

more frequently reported daily or almost daily cannabis use, suggesting the need for the provision of strains that optimize pain management while limiting adverse effects (i.e. low THC) (27).

Our findings demonstrate that medicinal programs are providing strains that are comparable in potency to those offered by recreational dispensaries. This lack of difference was also apparent when comparing medicinal and recreational programs within the same region. No difference in THC concentrations was observed between medicinal and recreational programs in the same state, neither when medicinal and recreational programs exist in different dispensaries (WA) nor when they exist in the same dispensary in different menus (CO). These findings suggest that, no matter how compared, there appears to be no clinically meaningful difference between medicinal and recreational cannabis potencies across the country.

We did not map concentrations in all dispensaries in all U.S. states where cannabis is legalized for either medical treatment of pain or recreational use; however, our study covers the two regions where most states have such programs. In contrast to smaller yet well-designed studies, our study covers Western and Northeastern regions from the U.S. and 8,505 cannabis strains across 653 dispensaries (15). Therefore, our results are likely a fair representation of the potency of online products offered in dispensaries across the U.S. It is challenging to generalize our findings to states not included in our study given the difference in legal requirements, size of state, population, and other demographics may drive the production and dispensing patterns in each state. However, our findings appear to be consistent and surprisingly uniform within and between the Western and Northeastern regions, which comprise very diverse geographic and demographic size, and differ widely in their cannabis program legal requirements. Our study indicates that both recreational and medicinal programs in various and diverse regions of the country are contributing to and reflecting the national trend towards increasing potency of cannabis.

While few dispensaries indica[illegible]e how their reported THC concentrations were measured or estimated, reported concentrations appear to be comparable to those measured in common strains from dispensaries located in CO,

CA, and WA. The average THC concentration in these products were consistently above 15% (28). Similarly, our data are in line with THC and CBD tested in samples seized by the DEA in 2015 (29) and 2017 (18), with an average THC of 20% and 17.8% respectively. The results of our study appear to be in agreement with such results, assuming that the trend toward higher concentrations of THC and predominance of high potency cannabis strains continued just as it has since the 1980s (19,20). Average CBD content of strains seized in 2017 was 0.15%, compared to 0.41% in 2008, resulting in an increase in the THC/CBD ratio from 23 in 2008 to 104 in 2017 (18). Our study identified a trend in which an increase in THC was accompanied by a decrease in CBD. This trend is concerning, as CBD can counteract THC-induced paranoia, memory impairment and positive psychotic symptoms and may provide some therapeutic benefit on its own (14,30,31). Indeed, high THC and low CBD is associated with higher risk of psychotic issues, memory problems, and dependence (32).

Although consistent with other cannabinoid concentrations reported throughout the literature, no universal standards for laboratory testing exist and previous studies have demonstrated the tendency for some laboratories to consistently report higher cannabinoid concentrations than their counterparts (16). Regardless of the method or laboratory used to measure THC content, the average THC concentration in all cases has been reported to be above 15% since 2014 (16). Thus, the THC content marketed online is consistent with reports in which THC was actually measured, suggesting a high level of concordance of our data with the existing literature, and suggesting the degree of inaccuracy of our data is similar to these studies. In addition to discrepancies between laboratories, a recent study demonstrated that CBD products advertise an accurate CBD concentration only 31% of the time and unlabeled THC was detected in about 21% of strains (33). Such results suggest a need for regulations that improve transparency in regard to the location and methods by which each product was tested.

The accuracy, or lack thereof, of labeled cannabinoid content of legal cannabis products poses a concern over patient's misconceptions regarding the potency of strains they consume. Such misconceptions lead to either unintended adverse effects due to use of a higher potency strain than anticipated, or to a perception of treatment failure due to use of a lower potency strain than expected. Although they may not represent completely accurate

cannabinoid concentrations, the cannabinoid content documented in our study are representative of what a patient would assess prior to selecting a strain of cannabis for medical use. As mentioned before, online information is a potent tool to promote a favorable attitude towards high potency cannabis for medicinal purposes (5,6), namely for the treatment of chronic pain. However, our data suggests that medicinal programs in the U.S. are not using scientific evidence to develop a legal framework for the safe provision of medicinal cannabis. While these states do offer strains amenable to medicinal use (THC <5-10%), these products are not what consumers primarily purchase. A Washington state cannabis potency and prices study demonstrated that flowers with THC > 15% accounted for over 90% of sales while flowers with THC <10% accounted for about 2% of expenditures between 2014 and 2016 (17). This evidence suggests that having some products suitable for medicinal use is not sufficient to provide safe treatment to patients seeking help in medical cannabis dispensaries, perhaps in part due to online advertisement of primarily high potency products. We recognize that other factors could be at play in the selection of high potency cannabis in medicinal programs. For one, more potent products are better regarded in recreational cannabis practices (12). Furthermore, since the price of a product increases with increasing THC concentration, revenue could contribute to budtender recommendations (17,34). In line with this assumption, a California study shows that over 40% of budtenders view medical decision-making as "less important" when making a recommendation (35). In addition, 70% of all dispensary staff reported that a lack of knowledge served as a barrier to making a medical recommendation (35). This trend is exacerbated by the fact that less than half of dispensaries across the country advise patients of potential side effects and even fewer warn of potential contraindications, while many promote ill-supported medical benefits (15).

The combination of these factors with our findings on online offering sets the grounds for policy makers and regulatory agencies to take action and guide towards the adoption of evidence based practices. A necessary first step for new regulatory policies is a clear differentiation between products used in medical versus recreational cannabis programs. States with comprehensive medicinal marijuana programs do not often pass legislation limiting the strength of cannabis on the market (36). Legislators should consider stricter regulations than are currently in place in order to deliver strains more amenable for pain and provide a safer health care service.

Regulations may include the addition of staff with proper training in pharmacology, patient counseling, and continual education in public health care. More collaboration among health professionals, scientists in pharmacology and pain specialists is needed to aid in the development of a more suitable legal framework for the provision and promotion of medicinal cannabis.

In conclusion, our findings suggest that medicinal programs are operating in a similar fashion to recreational programs based on the products they offer online (high THC/low CBD), which are not adequate for medical use and could contribute to risky misconceptions towards medicinal cannabis. To combat this, states might consider collaboration with healthcare professionals to develop a more suitable legal framework for safe medicinal cannabis use across the United States, which could serve as a model to other countries when considering medical cannabis legalization.

Acknowledgments

The authors would like to acknowledge the Department of Anesthesiology at Wake Forest University School of Medicine for funding.

Disclosures

The authors, Mary Catherine Cash, Katharine Cunnane, Chuyin Fan, and E. Alfonso Romero-Sandoval have no financial conflicts of interest to disclose.

References

1. Boehnke KF, Scott JR, Litinas E, Sisley S, Williams DA, Clauw DJ. Pills to Pot: Observational Analyses of Cannabis Substitution Among Medical Cannabis Users With Chronic Pain. J Pain . 2019 Jan 26;19(1):37.
2. Bradford AC, Bradford WD, Abraham A, Adams GB. Association between US state medical cannabis laws and opioid prescribing in the medicare part D population. JAMA Intern Med. 2018 May;178(5):667–73.

3. Vigil JM, Stith SS, Adams IM, Reeve AP. Associations between medical cannabis and prescription opioid use in chronic pain patients: A preliminary cohort study. Vrana KE, editor. PLoS One. 2017 Nov 16;12(11):e0187795.

4. Baron EP, Lucas P, Eades J, Hogue O. Patterns of medicinal cannabis use, strain analysis, and substitution effect among patients with migraine, headache, arthritis, and chronic pain in a medicinal cannabis cohort. J Headache Pain. 2018 Dec 24;19(1):37.

5. Caputi TL, Leas EC, Dredze M, Ayers JW. Online Sales of Marijuana: An Unrecognized Public Health Dilemma. Am J Prev Med. 2018 May;54(5):719–21.

6. Lewis N, Sznitman SR. Engagement with medical cannabis information from online and mass media sources: Is it related to medical cannabis attitudes and support for legalization? Int J Drug Policy. 2019 Feb 21

7. Capler R, Walsh Z, Crosby K, Belle-Isle L, Holtzman S, Lucas P, et al. Are dispensaries indispensable? Patient experiences of access to cannabis from medical cannabis dispensaries in Canada. Int J Drug Policy. 2017 Sep

8. FDA. FDA warns company marketing unapproved cannabidiol products with unsubstantiated claims to treat cancer, Alzheimer's disease, opioid withdrawal, pain and pet anxiety. Press Announcement. 2019 Jul 23

9. Wallace MS, Marcotte TD, Umlauf A, Gouaux B, Atkinson JH. Efficacy of Inhaled Cannabis on Painful Diabetic Neuropathy. J Pain . 2015 Jul;16(7):616–27.

10. Wilsey B, Marcotte T, Deutsch R, Gouaux B, Sakai S, Donaghe H. Low-dose vaporized cannabis significantly improves neuropathic pain. J Pain . 2013 Feb;14(2):136–48.

11. Andreae MH, Carter GM, Shaparin N, Suslov K, Ellis RJ, Ware MA, et al. Inhaled Cannabis for Chronic Neuropathic Pain: A Meta-analysis of Individual Patient Data. J Pain. 2015 Dec;16(12):1221–32.

12. Romero-Sandoval EA, Fincham JE, Kolano AL, Sharpe BN, Alvarado-Vázquez PA. Cannabis for Chronic Pain: Challenges and Considerations. Pharmacother J Hum Pharmacol Drug Ther . 2018 Jun;38(6):651–62.

13. National Academies of Sciences, Engineering, Medicine. The Health Effects of Cannabis and Cannabinoids . Washington, D.C.: National Academies Press; 2017.

14. Englund A, Morrison PD, Nottage J, Hague D, Kane F, Bonaccorso S, et al. Cannabidiol inhibits THC-elicited paranoid symptoms and hippocampal-dependent memory impairment. J Psychopharmacol . 2013 Jan;27(1):19–27.

15. Cavazos-Rehg PA, Krauss MJ, Cahn E, Lee KE, Ferguson E, Rajbhandari B, et al. Marijuana Promotion Online: an Investigation of Dispensary Practices. Prev Sci . 2019 Feb 9;20(2):280–90.

16. Jikomes N, Zoorob M. The Cannabinoid Content of Legal Cannabis in Washington State Varies Systematically Across Testing Facilities and Popular Consumer Products. Sci Rep . 2018 Mar 14;8(1):4519.

17. Smart R, Caulkins JP, Kilmer B, Davenport S, Midgette G. VARIATION IN CANNABIS POTENCY & PRICES IN A NEWLY-LEGAL MARKET: Evidence from 30 million cannabis sales in Washington State. Addiction . 2017 Dec;112(12):2167–77.

18. Chandra S, Radwan MM, Majumdar CG, Church JC, Freeman TP, ElSohly MA. New trends in cannabis potency in USA and Europe during the last decade (2008–2017). Eur Arch Psychiatry Clin Neurosci. 2019 Feb;269(1):5–15.

19. ElSohly MA, Ross SA, Mehmedic Z, Arafat R, Yi B, Banahan BF. Potency trends of delta9-THC and other cannabinoids in confiscated marijuana from 1980-1997. J Forensic Sci. 2000 Jan;45(1):24–30.

20. ElSohly MA, Mehmedic Z, Foster S, Gon C, Chandra S, Church JC. Changes in cannabis potency over the last 2 decades (1995-2014): Analysis of current data in the United States. Biol Psychiatry. 2016 Apr 1;79(7):613–9.

21. Monte AA, Shelton SK, Mills E, Saben J, Hopkinson A, Sonn B, et al. Acute Illness Associated With Cannabis Use, by Route of Exposure. Ann Intern Med. 2019 Apr 16;170(8):531.

22. Forti M Di, Quattrone D, Freeman TP, Tripoli G, Gayer-Anderson C, Quigley H, et al. The contribution of cannabis use to variation in the incidence of psychotic disorder across Europe (EU-GEI): a multicentre case-control study. 2019

23. Arterberry BJ, Treloar Padovano H, Foster KT, Zucker RA, Hicks BM. Higher average potency across the United States is associated with progression to first cannabis use disorder symptom. Drug Alcohol Depend. 2019 Feb 1;195:186–92.

24. Freeman TP, Winstock AR. Examining the profile of high-potency cannabis and its association with severity of cannabis dependence. Psychol Med . 2015 Nov 27;45(15):3181–9.

25. Volkow ND, Baler RD, Compton WM, Weiss SRBB, Longo DL, Volkow ND, et al. Adverse Health Effects of Marijuana Use. N Engl J Med . 2014 Jun 5;370(23):2219–27.

26. Freeman TP, van der Pol P, Kuijpers W, Wisselink J, Das RK, Rigter S, et al. Changes in cannabis potency and first-time admissions to drug treatment: a 16-year study in the Netherlands. Psychol Med. 2018 Oct 31;48(14):2346–52.

27. Lin LA, Ilgen MA, Jannausch M, Bohnert KM. Comparing adults who use cannabis medically with those who use recreationally: Results from a national sample. Addict Behav. 2016 Oct;61:99–103.

28. Vergara D, Bidwell LC, Gaudino R, Torres A, Du G, Ruthenburg TC, et al. Compromised External Validity: Federally Produced Cannabis Does Not Reflect Legal Markets. Sci Rep. 2017 Apr 19;7(1):46528.

29. WHO. The health and social effects of nonmedical cannabis use. Wayne Hall MR and VP, editor. WHO Document Production Services; 2016.

30. Iffland K, Grotenhermen F. An Update on Safety and Side Effects of Cannabidiol: A Review of Clinical Data and Relevant Animal Studies. Cannabis Cannabinoid Res . 2017 Jan;2(1):139–54.

31. Corroon J, Phillips JA. A Cross-Sectional Study of Cannabidiol Users. Cannabis cannabinoid Res. 2018;3(1):152–61.

32. Wilson J, Freeman TP, Mackie CJ. Effects of increasing cannabis potency on adolescent health. Lancet Child Adolesc Heal. 2019 Feb;3(2):121–8.

33. Bonn-Miller MO, Loflin MJE, Thomas BF, Marcu JP, Hyke T, Vandrey R. Labeling Accuracy of Cannabidiol Extracts Sold Online. JAMA. 2017 Nov 7;318(17):1708–9.

34. Hunt P, Pacula RL. Early Impacts of Marijuana Legalization: An Evaluation of Prices in Colorado and

Washington. J Prim Prev. 2017 Jun 29;38(3):221–48.

35. Peiper NC, Gourdet C, Meinhofer A, Reiman A, Reggente N. Medical Decision-Making Processes and Online Behaviors Among Cannabis Dispensary Staff. Subst Abus Res Treat . 2017 Jan 21;11:1–9.

36. Klieger SB, Gutman A, Allen L, Liccardo Pacula R, Ibrahim JK, Burris S, et al. Mapping medical marijuana: State laws regulating patients, product safety, supply chains and dispensaries, 2017. Addiction. 2017 Dec;112(12):2206–16.

Figures Legends

Figure 1. Average and range of percent THC (A) and percent CBD (B) in legalized medicinal or recreational dispensaries in the Northeastern and Western regions of the United States. Herb cannabis products offered in dispensaries from states with medicinal only (Med; ME, MA, NH, RI, VT, and NM) and recreational and medicinal (Rec; CA, CO, and CA) programs were included for analysis and represented individually as circles. Data are presented as mean ± SD. *P<0.05 vs. medicinal, by Welch's t test.

Figure 2. Average and range of percent THC (A) and percent CBD (B) per state. Percent THC and CBD was obtained from dispensary websites for herb cannabis products in the surveyed states and plotted as circles. States with medicinal only (Med; black circles) and recreational and medicinal (Rec; grey circles) programs were compared. Data are presented as mean ± SD. *P<0.05 vs. comparator state (linked lines), arrows indicate the state compared vs. the comparator states linked with a black filled circle (CO and WA). One-way ANOVA and Tukey's multiple comparison test.

Figure 3. Percent of strains with <5% THC (blue), ≥5%≤10% THC (purple), >10≤15% THC (yellow) and >15% THC (green) in all surveyed states. The total number of products analyzed per state is presented at the bottom of every graph (state) with medicinal programs (ME, VT, NH, RI, MA, and NM) or medicinal and recreational programs (CO, WA, and CA).

Figure 4. Average and range of percent THC at different strength product categories based on THC content: <5% THC (A), ≥5≤10% THC (B), >10≤15% THC (C), and >15% THC (D). Every herb cannabis product in the surveyed states is plotted as an open circle. States with medicinal only (Med; black circles) and recreational and medicinal (Rec; grey circles) programs were compared. Data are presented as mean ± SD. *P<0.05 vs. comparator state (linked lines), arrows indicate the state compared vs. the comparator states linked with a black filled circle (NH, CO and WA). One-way ANOVA and Tukey's multiple comparison test.

Figure 5. Average and range of percent CBD at different strength product categories based on THC content: <5% THC (A), ≥5≤10% THC (B), >10≤15% THC (C), and >15% THC (D). Every herb cannabis product in the surveyed states is plotted as an open circle. States with medicinal only (Med; black circles) and recreational and medicinal (Rec; grey circles) programs were compared. Data are presented as mean ± SD. *P<0.05 vs. comparator state (linked lines). One-way ANOVA and Tukey's multiple comparison test.

Figure 6. Average and range of percent THC (A) and percent CBD (B) in CO and WA medicinal and recreational programs. Data for CO was obtained from the respective medicinal or recreational menus offered in the same dispensary, while data for WA was obtained from either medicinal dispensaries or recreational dispensaries, which exist as independent entities. Herb cannabis products and their THC and CBD percent were included for analysis and represented individually as circles. *P<0.05 vs. comparator state (linked lines). One-way ANOVA and Tukey's multiple comparison test.

Figure 7. Percent of strains with <5% THC (blue), ≥5%≤10% THC (purple), >10≤15% THC (yellow) and >15% THC (green) in WA medicinal or recreational dispensaries (top panel, WA Medical and WA Recreational), and CO medicinal or recreational menus (bottom panel, CO Medical and CO Recreational). The total number of products analyzed per program is presented at the bottom of every graph (state and program).

Figure 8. Average and range of percent THC at different strength product categories based on THC content: <5% THC (A), ≥5≤10% THC (B), >10≤15% THC (C), and >15% THC (D) in CO recreational or medicinal menus and WA recreational or medicinal dispensaries. Every herb cannabis product in the surveyed states and programs are plotted as open circles. Medicinal programs (Med; black circles) and recreational programs (Rec; grey circles) were compared. Data are presented as mean ± SD. *P<0.05 vs. comparator state (linked lines). One-way ANOVA and Tukey's multiple comparison test.

Figure 9. Average and range of percent CBD at different strength product categories based on THC content: <5% THC (A), ≥5≤10% THC (B), >10≤15% THC (C), and >15% THC (D) in CO recreational or medicinal menus and WA recreational or medicinal dispensaries. Every herb cannabis product in the surveyed states and programs are plotted as open circles. Medicinal programs (Med; black circles) and recreational programs (Rec; grey circles) were compared. Data are presented as mean ± SD. *P<0.05 vs. comparator state (linked lines). One-way ANOVA and Tukey's multiple comparison test.

Tables

Table 1. Significant comparisons of average THC concentration (in parenthesis) in all sampled states. States in rows were compared with respective states in columns and the P values are reported. A P<0.05 was considered statistically significant. NS = not statistically significant.

	VT (15.2% ± 4.3)	ME (17.6% ± 4.4)	MA (19.4% ± 6.0)	NM (19.1% ± 6.3)	CA (19.1% ± 6.3)
CO (21.5 ± 5.6)	<0.0001	0.002	<0.0001	<0.0001	<0.0001
WA (21.7 ± 6.0)	<0.0001	0.0008	<0.0001	<0.0001	<0.0001
NH (20.3 ± 7.2)	0.01	NS	NS	NS	NS

Table 2. Significant comparisons of average THC concentration (in parenthesis) in the >15% THC category in all sampled states. States in rows were compared with respective states in columns and the P values are reported. A P<0.05 was considered statistically significant. NS = not statistically significant.

	VT (17.5% ± 1.0)	MA (21.5% ± 3.4)	NM (20.92% ± 3.8)	CA (20.62% ± 5.5)
CO (22.7% ± 4.2)	0.001	0.006	<0.0001	<0.0001
WA (22.5% ± 5.1)	0.002	0.03	<0.0001	<0.0001
NH (22.8% ± 4.0)	0.003	NS	0.02	0.003
VT (17.5% ± 1.0)	NS	0.05	NS	NS

Figure 1

Click here to download Figure Fig 1-Global THC and CBD med and rec.tiff

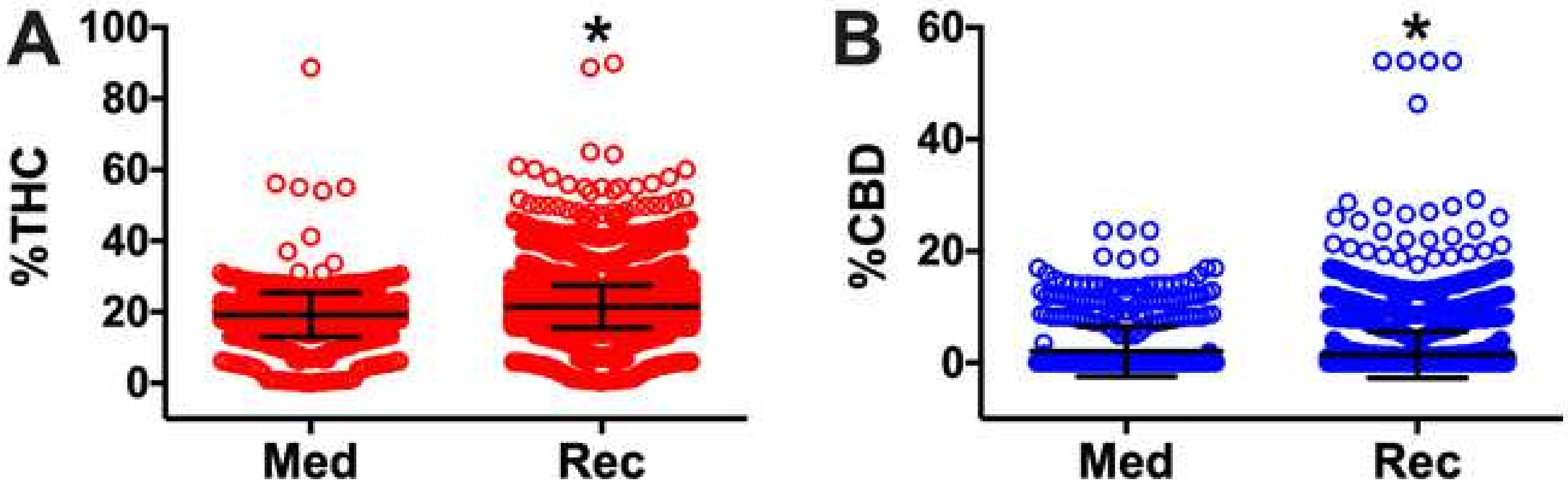

Figure 2

Click here to download Figure Fig 2 -Global THC and global CBD split by state.tiff

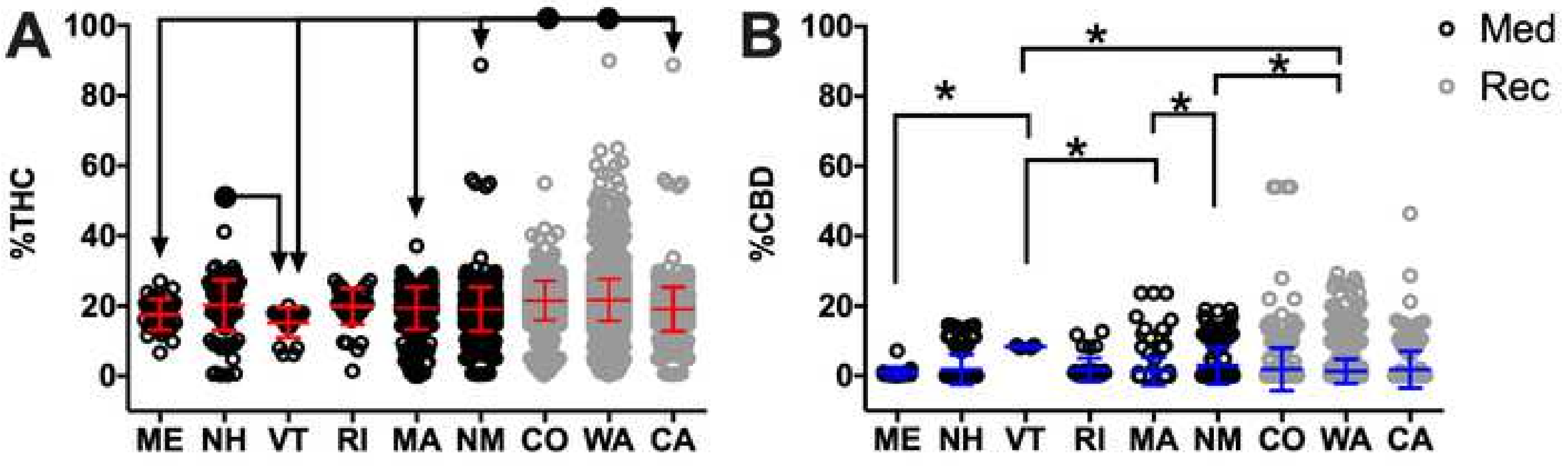

Figure 3

Click here to download Figure Fig 3 -Pie Charts.tiff

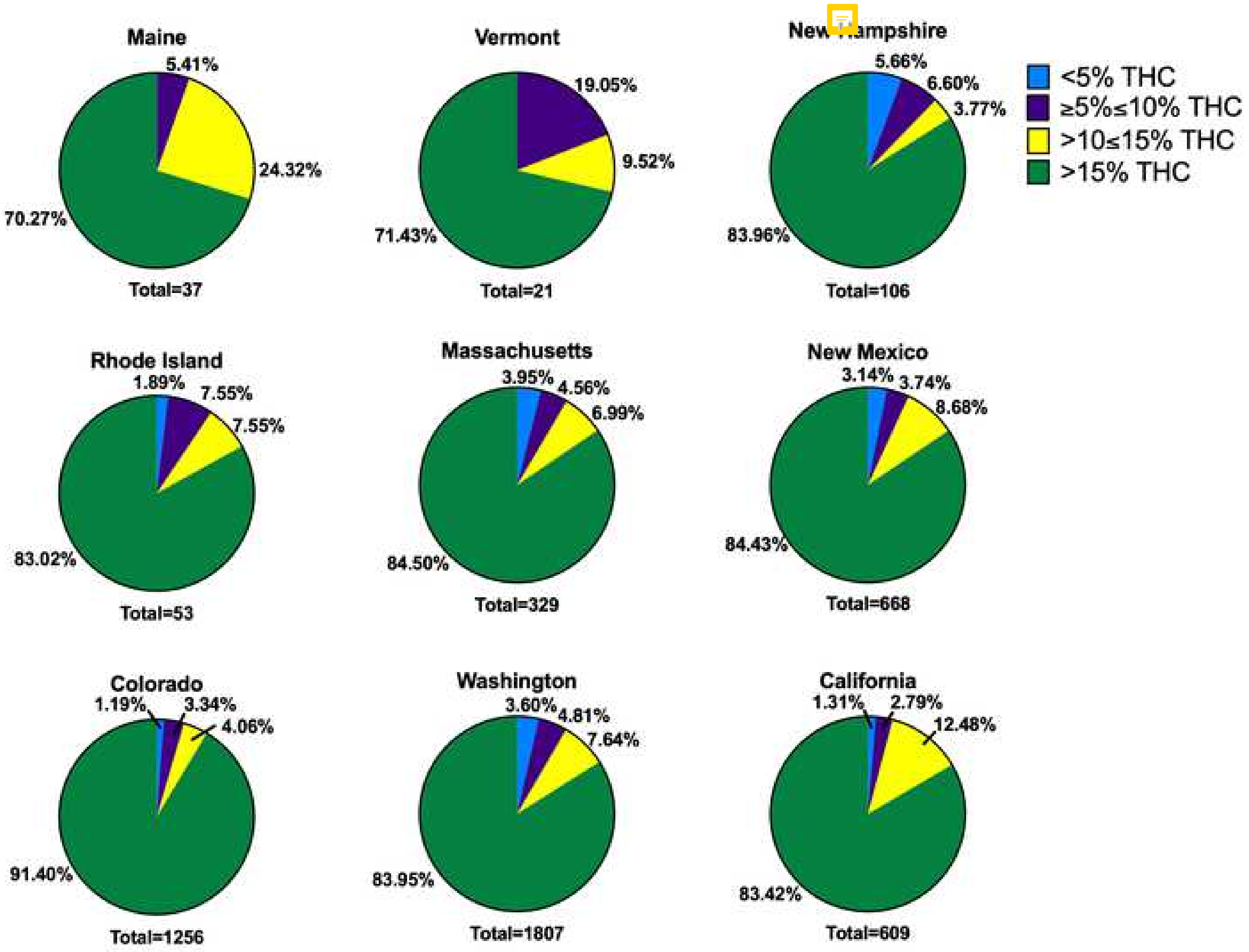

Figure 4

Click here to download Figure Fig 4 -THC split by percent.tiff

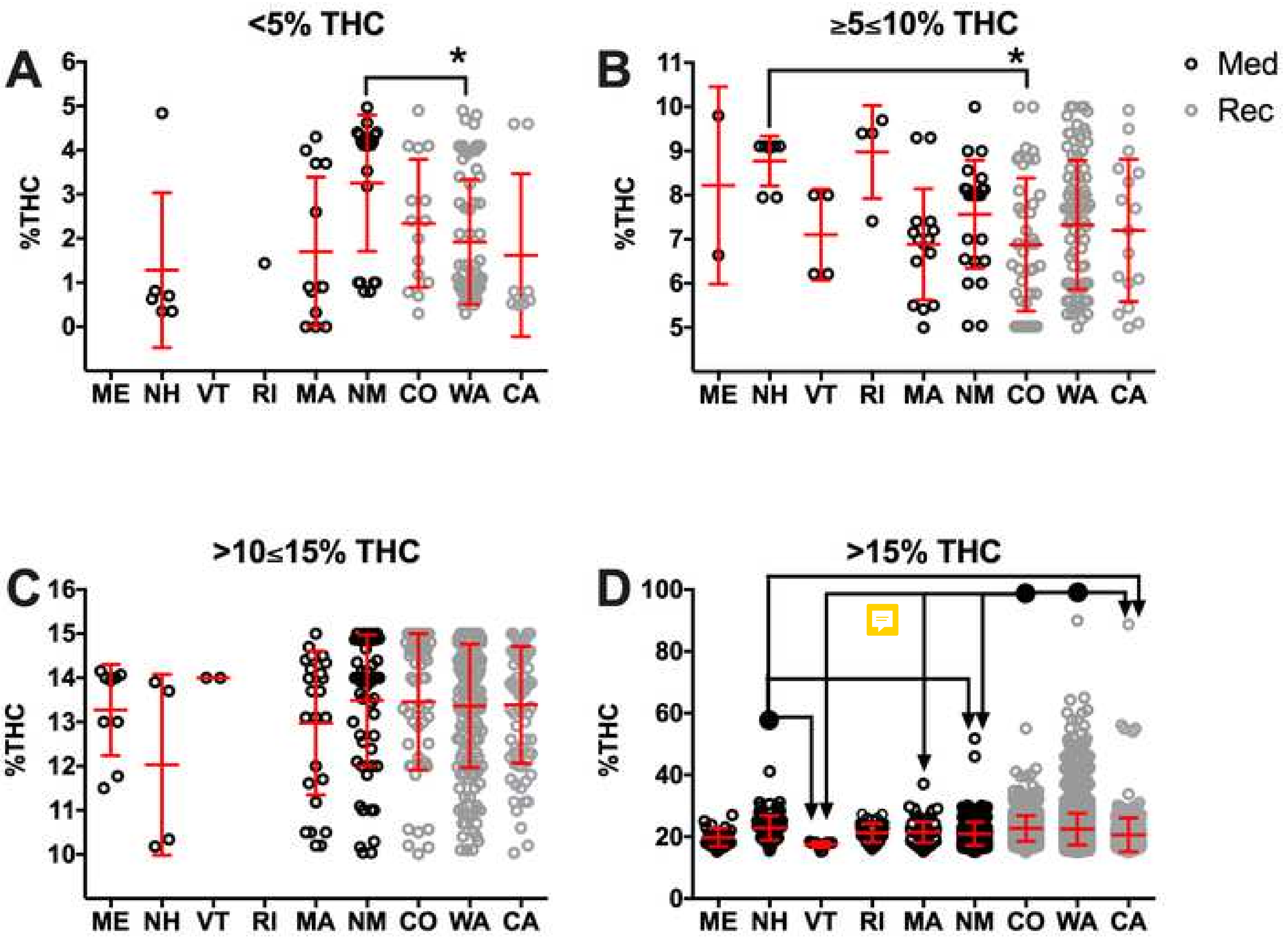

Figure 5 Click here to download Figure Fig 5 -CBD split by percent.tiff

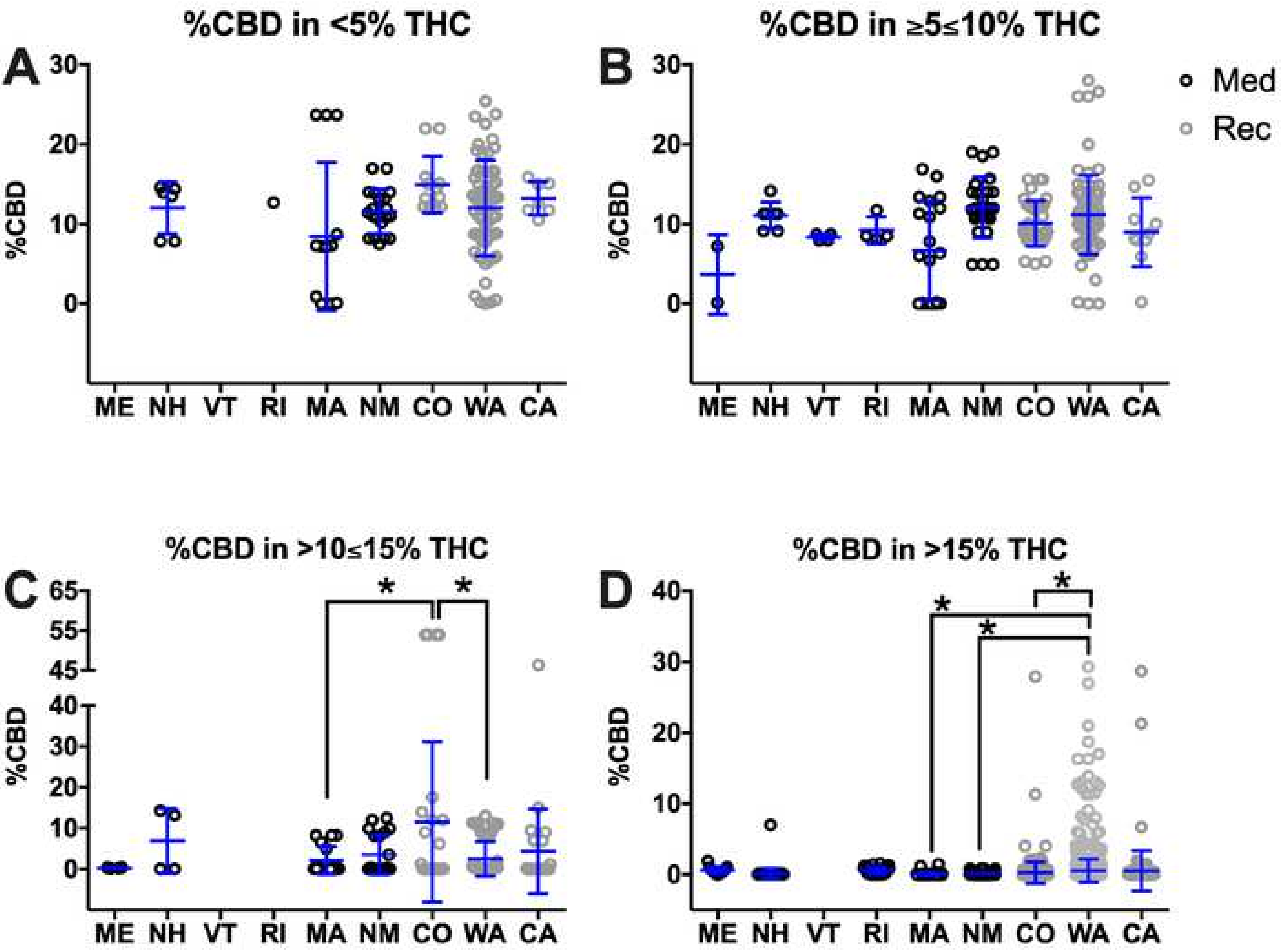

Figure 6

Click here to download Figure Fig 6 -within state thc and cbd.tiff

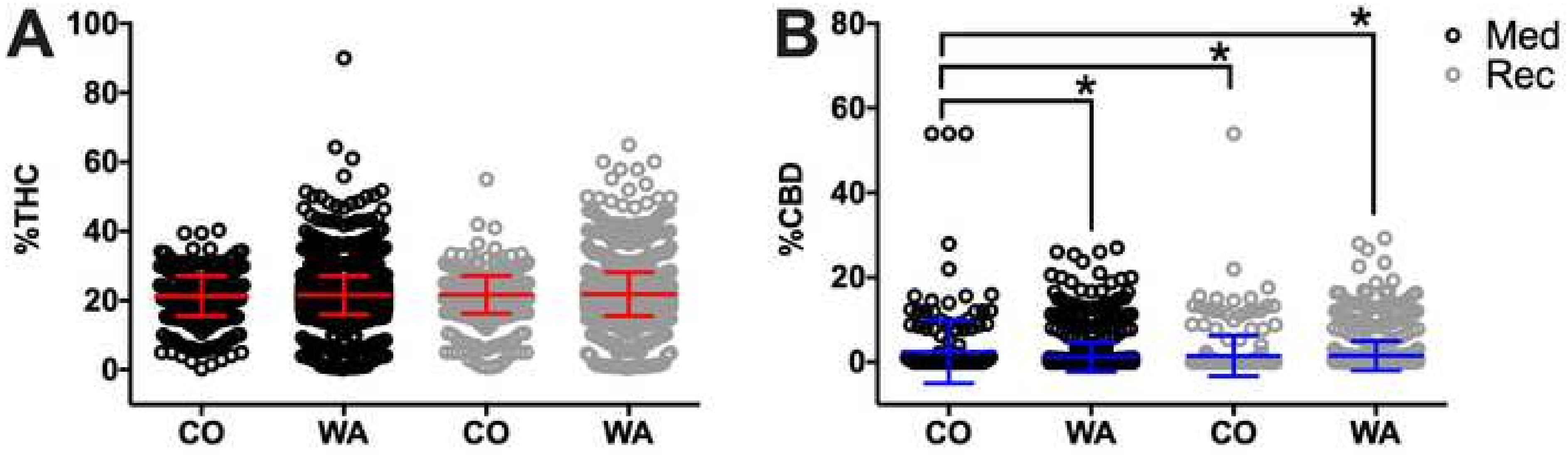

Figure 7 Click here to download Figure Fig 7 -WA and CO med vs rec pie charts.tiff

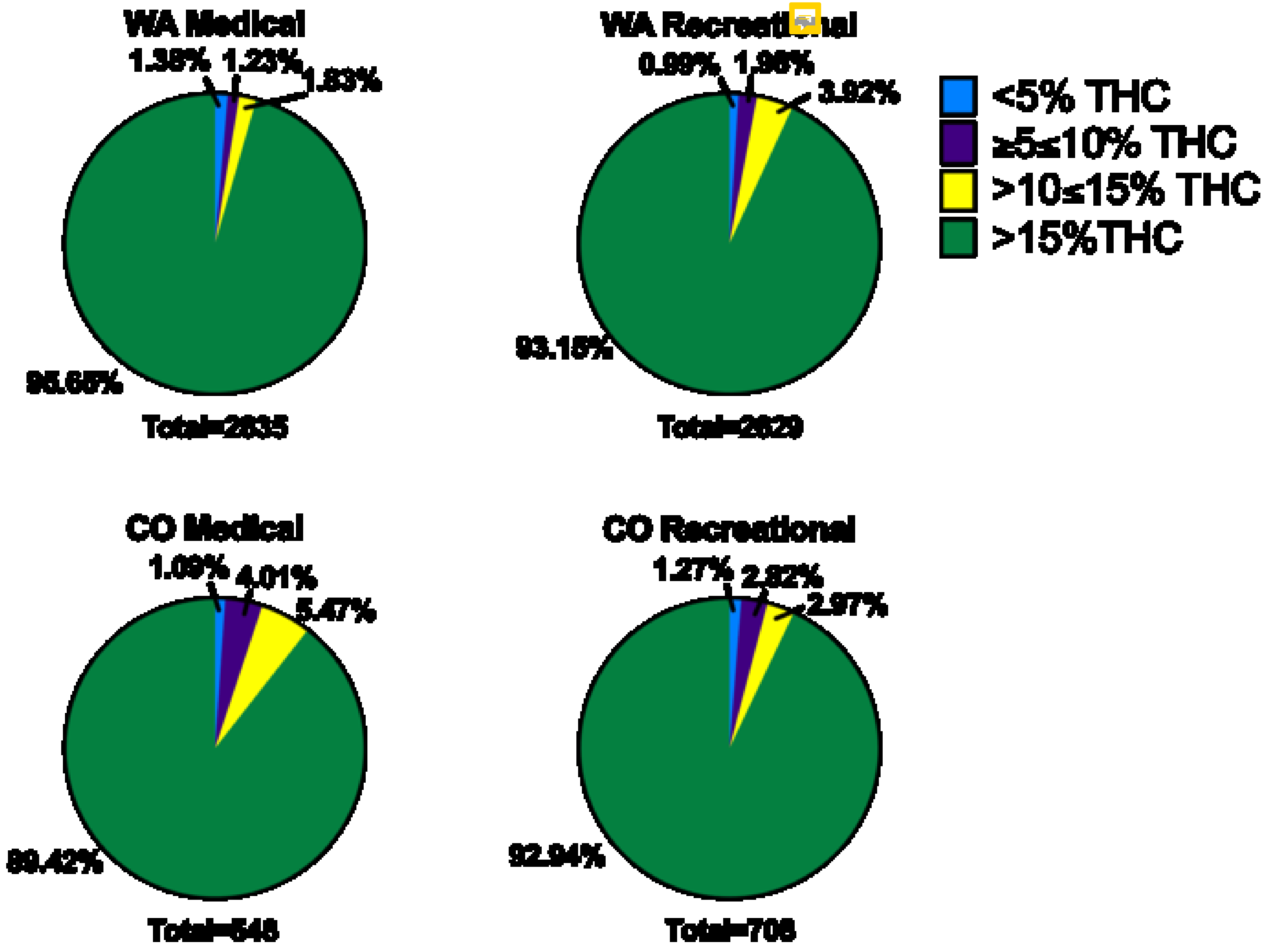

Figure 8

Click here to download Figure Fig 8 -THC by category by state CO_WA.tiff

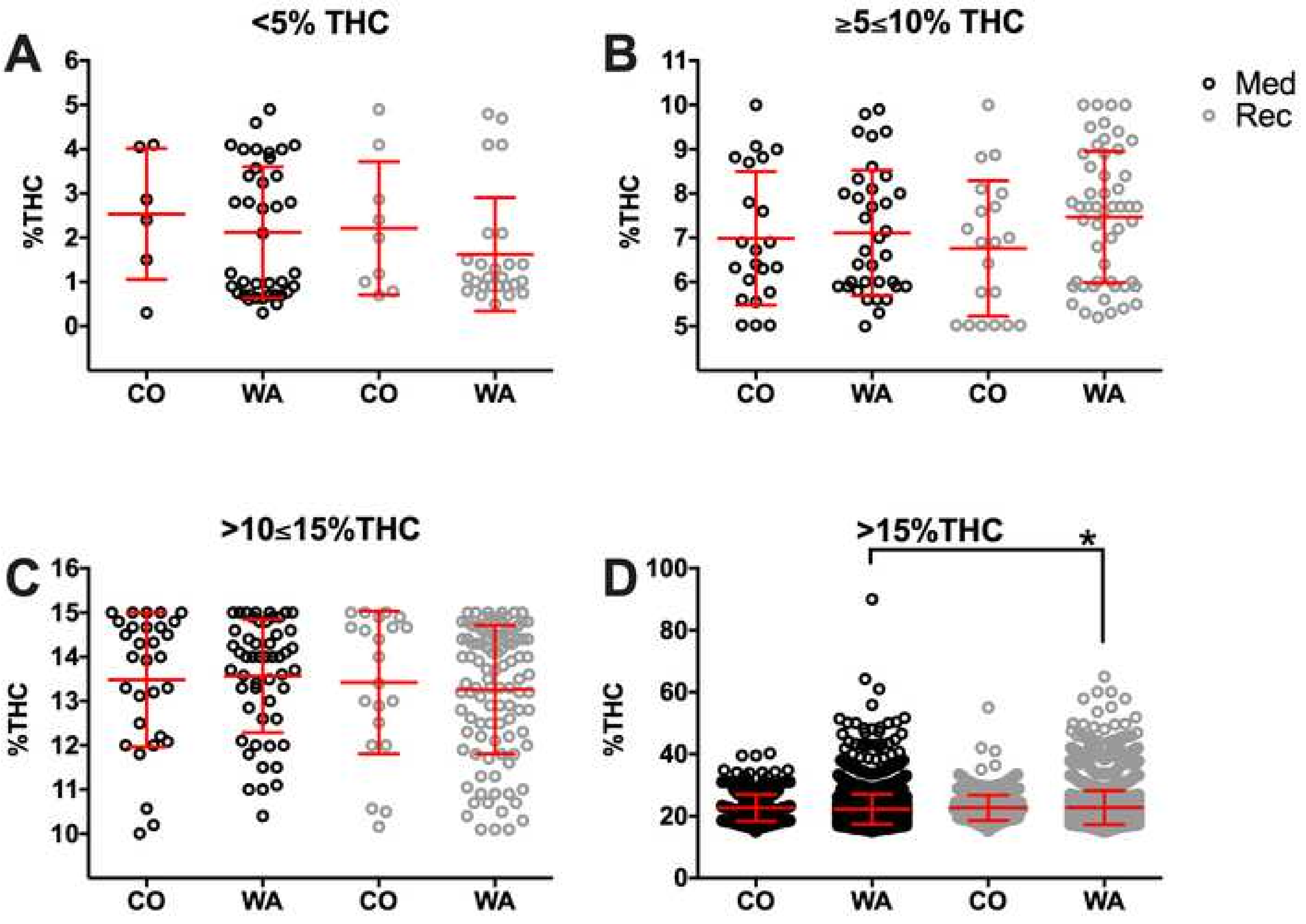

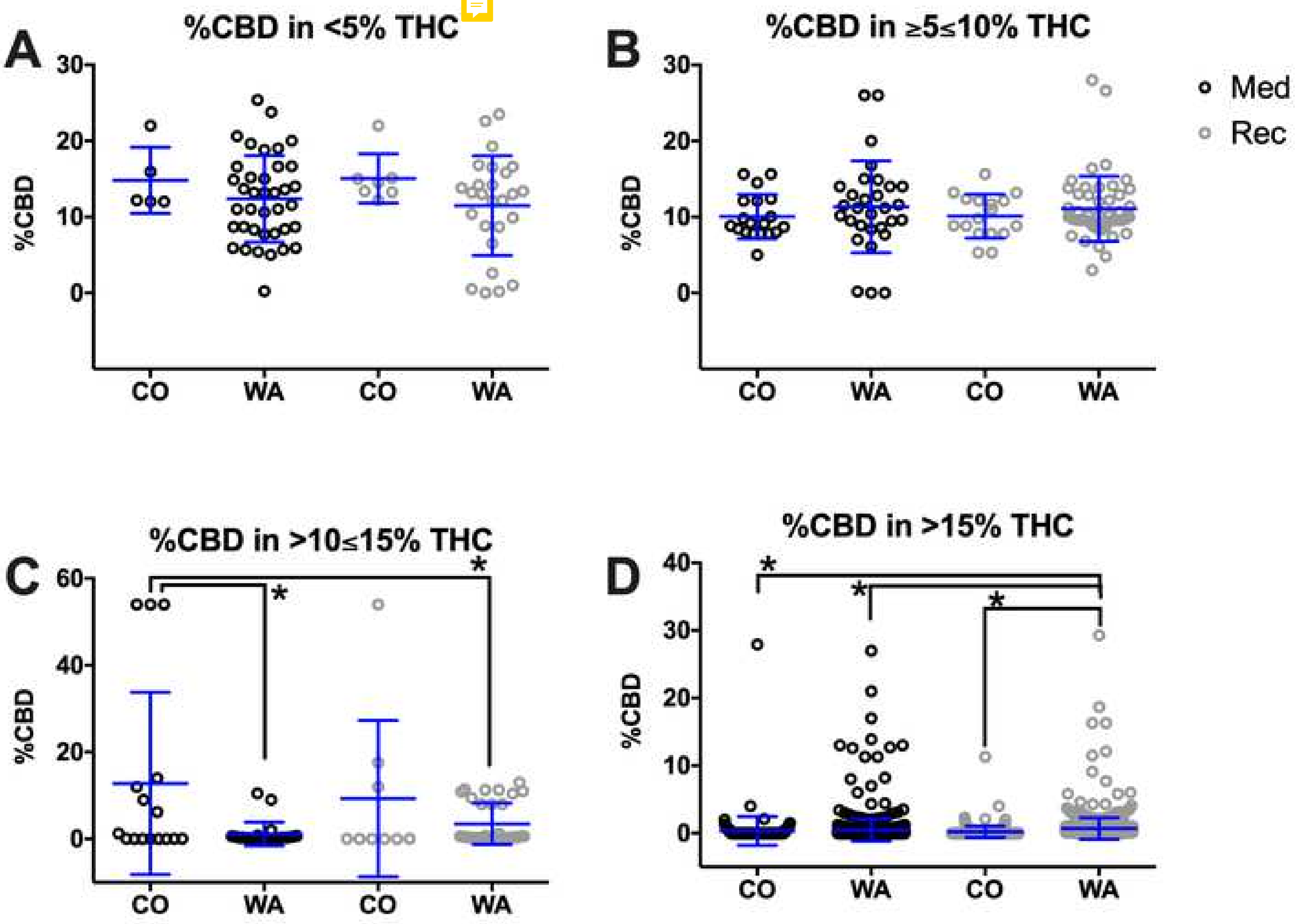
A
%CBD in <5% THC
%CBD
30
20
10
0
CO
WA
CO
WA
B
%CBD in ≥5≤10% THC
Med
Rec
C
%CBD in >10≤15% THC
60
40
*
D
%CBD in >15% THC
40

Click here to access/download

Supporting Information

Supplementary Info-2019.docx

Click here to access/download
Supporting Information
All data-THC CBD East.xlsx

Click here to access/download
Supporting Information
All data-THC CBD West.xlsx
