# Supplementary material for: Mapping cannabis potency in medical and recreational programs in the United States
Source: PLoS One. 2020 Mar 26;15(3):e0230167. doi: 10.1371/journal.pone.0230167 (PMC7098613; doi:10.1371/journal.pone.0230167)
Supplement: Attachment — Submitted filename: Plos One THC - Response to reviewers 2019.docx [file pone.0230167.s017.docx]

**Response to reviewers**

Please find below a point-by-point response to reviewers’ comments. Our responses and all changes in the manuscript are in blue font.

Journal Requirements:

When submitting your revision, we need you to address these additional requirements.

1) Please ensure that your manuscript meets PLOS ONE's style requirements, including those for file naming. The PLOS ONE style templates can be found at

<http://www.journals.plos.org/plosone/s/file?id=wjVg/PLOSOne_formatting_sample_main_body.pdf> and <http://www.journals.plos.org/plosone/s/file?id=ba62/PLOSOne_formatting_sample_title_authors_affiliations.pdf>

2) Please include in your methods section, the methods used to identify licensed cannabis dispensaries in the different states.

**Response:**

We have included the information requested. We have also moved specifics that were in supplementary information into the main Methods text. For clarity (and because the information is extensive) we do not reproduce the information added in the Methods in this letter. However, the new information is in blue font to facilitate the location of the edits.

3) Please include captions for your Supporting Information files at the end of your manuscript, and update any in-text citations to match accordingly. Please see our Supporting Information guidelines for more information: <http://journals.plos.org/plosone/s/supporting-information>.

**Response:**

This has been completed as indicated.

Reviewers' comments:

Reviewer's Responses to Questions

**Comments to the Author**

1. Is the manuscript technically sound, and do the data support the conclusions?

The manuscript must describe a technically sound piece of scientific research with data that supports the conclusions. Experiments must have been conducted rigorously, with appropriate controls, replication, and sample sizes. The conclusions must be drawn appropriately based on the data presented. 

Reviewer #1: Partly

Reviewer #2: Partly

Reviewer #3: Yes

2. Has the statistical analysis been performed appropriately and rigorously? 

Reviewer #1: Yes

Reviewer #2: Yes

Reviewer #3: Yes

3. Have the authors made all data underlying the findings in their manuscript fully available?

The [PLOS Data policy](http://www.plosone.org/static/policies.action#sharing) requires authors to make all data underlying the findings described in their manuscript fully available without restriction, with rare exception (please refer to the Data Availability Statement in the manuscript PDF file). The data should be provided as part of the manuscript or its supporting information, or deposited to a public repository. For example, in addition to summary statistics, the data points behind means, medians and variance measures should be available. If there are restrictions on publicly sharing data—e.g. participant privacy or use of data from a third party—those must be specified.

Reviewer #1: Yes

Reviewer #2: Yes

Reviewer #3: Yes

4. Is the manuscript presented in an intelligible fashion and written in standard English?

PLOS ONE does not copyedit accepted manuscripts, so the language in submitted articles must be clear, correct, and unambiguous. Any typographical or grammatical errors should be corrected at revision, so please note any specific errors here.

Reviewer #1: Yes

Reviewer #2: Yes

Reviewer #3: Yes

5. Review Comments to the Author

Please use the space provided to explain your answers to the questions above. You may also include additional comments for the author, including concerns about dual publication, research ethics, or publication ethics. (Please upload your review as an attachment if it exceeds 20,000 characters)

**Reviewer #1:**

This manuscript surveys and compares the reported THC and CBD concentrations found in cannabis distributed for medicinal versus recreational purposes across many US states. This is an extensive study accessing publicly available information from websites maintained by the cannabis distributors. They found that THC and CBD concentrations are generally very high and very variable. Recreational cannabis has slightly higher concentrations of THC and CBD with large state to state variability. This information is potentially important for the medical and scientific communities, however, the conclusions by the authors that access to low (and in their opinion more effective) concentration THC from medical distributors are an over interpretation of the findings. Further, determining actual concentrations of THC and CBD, while very difficult at this scale, and comparing to the reported values would be more enlightening. Below are specific comments on the manuscript:

**Comments:**

1. In the 2nd paragraph of the introduction, it is stated that an increase in THC content results in a loss of analgesic effects. However, the cited studies actually report superior analgesic effects from “high” THC content of ~7% THC products than from “low” or “medium” THC content of 1-4% THC products, as well as impaired performance on certain tests such as the completion time of the Paced Auditory Serial Addition test and Trail Making test. Further, these “high” THC product concentrations are in the middle range of the concentrations suggested to be efficacious for pain with minimal psychotropic effects in the next line. These statements and citations are inconsistent with respect to analgesic efficacy and adverse events.

**Response:**

We appreciate this remark and apologize for not being accurate in our statement. We have re-worded this part of the introduction, acknowledging the dose-dependent effect of THC as an analgesic, but recognizing that an increase in THC concentration also increases the risk of side effects and might result in an increase in pain, as demonstrated in experimental pain studies in humans. This new text reads as follows:

“Even though the analgesic effects of THC are dose dependent [16, 17], an increase in THC content (%THC for inhaled formulations) increases the frequency of adverse events [11, 14, 15], and might increase pain, as demonstrated in experimental pain studies in humans [17, 18].”

**Comments:**
2. Much more important than the concentration of cannabinoids in plant material, and not mentioned here or elsewhere, is the quantity of plant material per administration. The potency of plant material is far less important than determining the quantity of cannabinoids administered.

**Response:**

We appreciate the opportunity to clarify this point. Even though this reviewer’s comment sounds logical and has merit, the risk of toxicity or side effects produced by more potent products is higher than products with lower concentrations of active ingredients. In addition to this pharmacologic premise, smoking behavior, such as shorter but more frequent inhalations, has been shown to be a risk factor for cannabis dependence regardless of product potency. Thus, the availability of the least potent presentation to achieve a given therapeutic effect is a premise that is widely accepted by regulatory entities of pharmaceutical compounds. In fact, this is the rationale behind the regulation of over the counter medications with significantly less toxic liabilities or drugs with far less abuse potential than cannabis. We added these considerations in our discussion to further support the advocation that medicinal cannabis not rely on products used for recreational purposes that are more potent than what is needed to reduce pain. In fact, the scientific and medical community should question the existence of high potency products even in the legal recreational realm. The new text reads as follows:

“The combination of these factors with our findings on online offering sets the grounds for policy makers and regulatory agencies to take action and guide towards the adoption of evidence based practices. Regulatory bodies have already adopted the practice of overseeing medications deemed safe for over the counter (OTC) use at low doses but with serious dose-dependent adverse effects. Such is the case with ibuprofen, which can be obtained OTC at the low doses used for mild to moderate pain but requires a prescription for the higher doses used for rheumatoid arthritis and osteoarthritis. In 2014, the FDA concluded the need for a new OTC label that conveys the seriousness of this risk [51]. This is in stark contrast to the paucity of regulation regarding the labeling and advertising of the dose-dependent adverse risks of cannabis in legal programs in the U.S. Additionally, OTC medications that are considered safe but that convey the risk of abuse are often highly regulated, as is the case with pseudoephedrine. Interestingly, Uruguay regulates cannabis in a manner similar to how the U.S. regulates pseudoephedrine, requiring it be purchased in a pharmacy and limiting the amount that can be purchased in the course of a week and over a month [52]. Of note, the legal products offered in Uruguay have a THC concentration that is <10% to reduce the risk of dependence [53].

Limiting the availability of cannabis products for medical purposes to those with moderate THC concentrations (<10%) would reduce the risk of acute side effects and long-term undesirable outcomes. In line with this assumption, the availability of higher potency cannabis products has been linked to higher blood levels of THC in users that have been apprehended for driving under the influence of drugs [54]. In addition, it has been demonstrated under experimental conditions that when cannabis products of varying potency are made available to users who were asked to use sufficient amounts to achieve a given psychotropic state, the election of high potency products resulted in the exposure to higher amounts of THC [55]. It is logical and tempting to think that the amount of product that is consumed is more important than its potency and that cannabis users are likely to titrate the amount of product consumed based on its strength. Indeed, it has been shown that users of high potency cannabis inhale less volumes; however, they use larger amounts of cannabis and are therefore exposed to higher levels of THC compared to users of low potency products [56]. This shows that real titration does not exist when high potency products are used. These studies demonstrate that psychosocial variables are more relevant than pharmacological variables in cannabis use behavior [55, 56]. This, together with the fact that high THC exposure is a risk factor to develop cannabis use disorder and that cannabis smoking behavior is a predictive factor to the severity of cannabis dependence (regardless of THC exposure, [56]) warrants regulations that disallow high potency products for medicinal use. These concepts should be also considered within the recreational realm.”

**Comments:**

3. For the potency of cannabis products to be meaningful, the quantity of cannabis products consumed must also be known to determine the total cannabinoids administered. For high potency cannabis products to pose a greater health risk, they would have to be consumed in equal quantities as lower potency products, but no evidence of this is presented and this important qualification is never stated. Statements claiming that the potency of cannabis products per se pose a health risk should be removed or qualified to address this.

**Response:**

We completely agree with this comment, and we apologize for not being more thorough on this point. As mentioned above, there is scientific evidence that more potent cannabis products do not lead to decreased THC intake. In fact, it is quite the contrary, as the evidence indicates that these users are exposed to higher levels of THC, which poses a higher risk of acute intoxication and potential for long-term side effects. These comments have been included in the previous comment’s response.

**Comments:**

4. It is stated that data was collected only for cannabis flower and pre-rolls. However, the range of the reported data includes strains with approximately 90% THC by weight, or over 50% CBD by weight. These outliers are certainly concentrated cannabis extracts or formulations, not flower. This should be addressed and the data checked for accuracy.

**Response:**

We thank the reviewer for this remark. Our intention was to report what the online information includes in the dispensaries’ website. However, we agree with the reviewer that these concentrations could represent non herbal products. We revisited our data and notes, and have identified that some products in fact were herbal and infused with extracts. We removed such products from the analysis and graphs. We still include some products that have high THC concentrations because we did not find any indication that they were infused herbal products, even though they might be. Since our intention is to report what the consumer will see in these webpages, we believe that it is important to reflect what the websites actually include. We did remark in the figure legends that these high potency products might be infused products, but we do not have any evidence for such a claim. The following statement was included in Figures 1, 2, 4, 6, and 8.

“The outlier products in the graph might represent herbal products infused with concentrated THC or CBD extracts, however we did not observe such a claim in the studied websites where these products were offered.”

**Comments:**

5. At the end of paragraph 4 in the discussion, it is mentioned that strains higher in THC were lower in CBD. This inverse relationship is a reported result of selection for either cannabinoid, and the increase in high CBD and low THC cannabis strains is not mentioned here.

**Response:**

This is a pertinent comment. We have re-worded the references to our observations in regard to THC and CBD containing products. These comments now read as follows:

Paragraph 4 in the Discussion:

“Our study identified ~~a trend in which an increase in THC was accompanied by a decrease in CBD~~ a greater number of products with high THC content compared to fewer products with some CBD content. This trend is concerning, as the psychotic effects induced by cannabis is THC dose dependent [43, 44] and CBD has the potential to counteract THC-induced paranoia, memory impairment and positive psychotic symptoms and may provide some therapeutic benefit on its own [25, 45, 46]. ~~Indeed, high THC and low CBD is associated with higher risk of psychotic issues, memory problems, and dependence (Wilson, Freeman, & Mackie, 2019)~~.”

Conclusion in the Discussion:

“In conclusion, our findings suggest that medicinal programs are operating in a similar fashion to recreational programs based on the products they offer online (high THC), which are not adequate for medical use and could contribute to risky misconceptions towards medicinal cannabis.”

**Reviewer #2:**

The current manuscript entitled “Mapping cannabis potency in medical and recreational programs in the United States” addresses a very important issue in medical cannabis use in the US. An accretion of evidence supports the public health risks of the regular use of high-THC products; patients, healthcare providers, and policy makers would all benefit from enhanced attention to the risks involved with potent THC products. However, in order for the manuscript to be suitable for publication in PLOS, it needs significant revisions to the Introduction, Results, Figures, and Discussion.

**Comments:**

Major concerns:

1) The foundation of the manuscript is based on the assumption that the medically-acceptable phenotype of analgesic cannabis is a flower with 5-10% THC. Although the analgesic efficacy of low-potency flower has been demonstrated in the literature (which the authors properly cite), there is an over-reliance on this single study throughout the manuscript, and a failure to acknowledge the large body of evidence which also shows the analgesic efficacy of unknown cannabis phenotypes, as well as recent studies showing that THC potency is the strongest predictor of analgesia (Le et al. 2019, PMID 31519268): in other words, the exact opposite of the authors’ presumed medically-relevant phenotype. There is not yet enough high-quality evidence for the scientific/medical communities to agree upon what a medical cannabis phenotype is (that which has maximal efficacy and minimal side effects). That said, there is ample evidence to support the RISKS associated with THC potency: this is the stronger argument for the evidence presented within the manuscript. The introduction and conclusion should clearly state for the reader that there is likely a very wide therapeutic window (in terms of analgesia) for THC, but that the risks of chronic consumption at the upper limit of the therapeutic window may be detrimental (cannabis use disorder, hyperemesis, cognitive impairment etc.).

**Response:**

We thank the reviewer for the opportunity to clarify this point. We now provide 6 original studies that we consider high quality based on the fact that these are randomized controlled trials (which were included in reviews that we cited previously). These studies show that analgesic effects could be achieved in chronic pain conditions with rather low to moderate potency cannabis products (<5 or 10% THC) with minimal side effects. The references are as follows:

- Ware MA, Wang T, Shapiro S, Robinson A, Ducruet T, Huynh T,et al. Smoked cannabis for chronic neuropathic pain: a randomized controlled trial. CMAJ. 2010;182(14):E694–701. https://doi.org/10.1503/cmaj.091414.
- Wilsey B, Marcotte T, Deutsch R, Gouaux B, Sakai S, Donaghe H. Low-dose vaporized cannabis significantly improves neuropathic pain. J Pain. 2013;14(2):136–48. <https://doi.org/10.1016/j.jpain>. 2012.10.009.
- Wilsey B, Marcotte T, Tsodikov A, Millman J, Bentley H, Gouaux B, et al. A randomized, placebo-controlled, crossover trial of cannabis cigarettes in neuropathic pain. J Pain. 2008;9(6):506–21. https://doi.org/10.1016/j.jpain.2007.12.010.
- Abrams DI, Jay CA, Shade SB, Vizoso H, Reda H, Press S, et al. Cannabis in painful HIV-associated sensory neuropathy: a randomized placebo-controlled trial. Neurology. 2007;68(7):515–21. https://doi.org/10.1212/01.wnl.0000253187.66183.9c.
- Ellis RJ, Toperoff W, Vaida F, van den Brande G, Gonzales J, Gouaux B, et al. Smoked medicinal cannabis for neuropathic pain i n HIV: a randomiz e d , c r o s s o v e r c l i n i c a l t r i a l . Neuropsychopharmacology. 2009;34(3):672–80. https://doi.org/10.1038/npp.2008.120. 27.
- Wallace MS, Marcotte TD, Umlauf A, Gouaux B, Atkinson JH. Efficacy of inhaled cannabis on painful diabetic neuropathy. J Pain. 2015;16(7):616–27. https://doi.org/10.1016/j.jpain.2015.03. 008.

We added Le et al. 2019, PMID 31519268 study as a reference when referring to the cannabis dose dependent analgesic effects. However, we would like to make a remark on this point. We recognize that very high potency cannabis products might produce better analgesia, but there is no doubt that higher THC concentrations will also produce severely intoxicating effects in naïve-cannabis users, namely a large proportion of chronic pain patients. Therefore, we reason that there is no net benefit in using cannabis products that would put patients at a higher risk of severe acute intoxication because analgesia could be achieved with minimal side effects with low or moderate THC concentrations. In addition, there is scientific evidence that higher THC concentrations could result in an increase in pain, which is in contrast to the reviewer’s comment on the wide therapeutic window of cannabis for the treatment of pain. We included those references in the manuscript.

- W.C. Clark, M.N. Janal, P. Zeidenberg, G.G. Nahas **Effects of moderate and high doses of marihuana on thermal pain: A sensory decision theory analysis.** J Clin Pharmacol, 21 (1981), pp. 299S-310S
- M. Wallace, G. Schulteis, J.H. Atkinson, T.Wolfson, D. Lazzaretto, H. Bentley, B.Gouaux, I. Abramson**Dose-dependent effects of smoked cannabis on capsaicin-induced pain and hyperalgesia in healthy volunteers.** Anesthesiology, 107 (2007), pp. 785-796

The edited paragraph in the introduction now reads as follows:

“THC is the primary constituent of cannabis and is responsible for its analgesic and euphoric effects, as well as its adverse effects. Even though the analgesic effects of THC are dose dependent [16, 17], an increase in THC content (%THC for inhaled formulations) increases the frequency of adverse events [11, 14, 15], and might increase pain, as demonstrated in experimental pain studies in humans [17, 18]. An array of studies have demonstrated efficacy in pain reduction with minimal and tolerable intoxicating effects with THC concentrations lower than 5% or 10% [14, 15, 19-22].”

**Comments:**

2) Although the dataset is thorough and sufficient for analysis, extreme outlying values in THC potency warrant the inclusion of the median, in addition to the mean THC potencies found in each legal market. Furthermore, the presentation of the data in the figures is not particularly intuitive. Some of the figures are difficult to read, either because of overcrowding with unnecessary statistical comparisons, or low-resolution of images. The inclusion of potency histograms would be of great utility to the readership of PLOS. Also, in the Introduction the authors mention the importance of the ratio of CBD:THC, however this is never directly presented in the figures.

**Response:**

We appreciate this reviewer’s comment. We have made several changes as suggested by the reviewers:

1) We removed the statistical comparisons from the graphs and indicated them in supportive tables (S4, S5, and S7). This certainly resulted in a clearer representation of the data.
2) We apologize for the low quality of the original images (they were 300 dpi). We now include higher resolution images, i.e. 600 dpi.
3) In addition, we have created frequency histograms in which we combine all studied states and the frequency of products in a given THC or CBD concentration. These combined histograms are now part of Figure 2.
4) In addition, we also reproduce these frequency histograms in individual graphs for each state to add clarity, and these graphs are now in supportive information (S1 and S3 Figures).
5) We also constructed potency histograms for each state in which the potency of the products are shown in relation to every individual product. These histograms provide a visual representation of the total amount of products offered in each state and are part of in supportive information (S2 and S4 Figures).

We consider that this new data representation is more intuitive and easier to follow. We agree that the histograms provide an additional visual representation of our findings.

In regard to the comment on THC and CBD ratio has been removed from the introduction, and other references to their interaction have been re-worded to better reflect our data (see previous comments from other reviewers). The new phrase in the Introduction reads as follows:

“Thus, the concentration of THC and CBD seems to ~~play a crucial role in the interaction between the two cannabinoids, contributing~~  contribute to the overall psychotropic and therapeutic effects patients experience.”

**Comments:**

Minor comments:

There are several instances of inaccurate interpretations of the cited literature throughout the manuscript. There is also a heavy reliance upon meta-analyses, commentaries, and systematic reviews instead of primary research articles.

**Response:**

We have reviewed our manuscript and we confirmed we have some reviews, meta-analysis, reports, and institutional statements (FDA of NIDA). We believe that these citations are supportive to original research properly cited with these documents rather than the foundation of our interpretation. We provide justification to include these studies as follows:

- We cite two meta-analysis, one of which is a seminal study showing that inhaled cannabis is effective in the treatment of chronic pain (Andreae MH et al., J Pain 2015). This study is important because it does not mix oral cannabinoids with inhaled products; therefore, we consider it pertinent to our study, which focuses on herbal products used for inhalation administration. The other is the report of the National Academies of Sciences, and is the most comprehensive report of scientific evidence on the effects of cannabis. This report concludes that there is conclusive evidence that inhaled cannabis is effective to treat chronic pain, which is pertinent for our current study. These studies are cited alongside several original studies.
- We cite a review from our group (Romero-Sandoval, et al., 2018) because in that analysis we discuss the role of medicinal cannabis and how the recreational realm influences it. To our knowledge there is no other document that relates this in the context of chronic pain. We now cite this review in conjunction with a very recent publication (original research study) that supports our thesis, i.e. Shi Y et al., Int J Drug Policy 2019).
- We include a Press Communication from FDA in regard to unsubstantiated claims of cannabis products offered online, which support the study of our hypothesis and reflects the seriousness of online information that could be risky for users.
- We include a report from the World Health Organization in which the use of non-medical cannabis is evaluated and related to high potency products worldwide and in particular in the USA. This report is also cited along with other original research papers.
- We also include a NIDA statement in the New England Journal of Medicine on the serious side effects derived from the use of high potency cannabis. We consider this article to have major repercussions in the field given the concerns voiced by the organization and the impact of the journal. This article is accompanied by two original research articles on the topic.
- We cite a review on clinical studies addressing CBD and its safety and side effects (Iffland K and Grotenhermen F, Cannabis Cannabinoid Res 2017). This review is accompanied by other two original research articles.
- We removed the comment by Wilson J et al., Lancet Child Adolesc Heal 2019.

**Comments:**

The authors utilize colloquial language throughout the manuscript, which strongly warrants correction: the readers of PLOS are looking to the journal to provide the most up-to-date and scientifically accurate information about cannabis, which should not include the words “strain” “indica” or “sativa,” unless specifically discussing the mythology and scientific inaccuracies that these terms impart. Furthermore, the terms psychotropic and psychoactive warrant clear differentiation, as do the psychological phenomenon of euphoria and impairment.

**Response:**

We apologize for the use of some colloquial terms, such as “strain”, “indica”, or “sativa”. Our intention was to accurately reflect the information that the dispensaries’ websites use, hence the use of such terms. However, we agree with the reviewer’s comment since this could be distracting for readers. Instead, we use “chemotypes” or simply “products”. We believe that this terminology is more accurate.

Similarly, we agree with the reviewer’s comment regarding the definition of “psychotropic” and “psychoactive”. Consequently, we use “intoxicating effects”. The phrase in the introduction using those terms now reads as follows:

“The main components found in cannabis are tetrahydrocannabinol (THC), the constituent of cannabis responsible for inducing intoxicating effects, and cannabidiol (CBD), which is devoid of intoxicating effects.”

**Comments:**

By addressing these comments and providing a more detailed analysis of the data (median), and presenting this data in more intuitive figures, the readers of PLOS would undoubtedly benefit from the evidence the authors present here.

**Response:**

We thank the reviewer for identifying these points and for the positive comments on our study.

**Reviewer #3:**

This manuscript reports the results of a cannabis potency surveyed done using dispensaries websites and how it relates to their medical and recreational use. It is well accepted that recreational cannabis-based produces are driven by high content in THC, whereas medical products contain lower amounts of THC and are often supplemented with CBD. The study is well designed, and the interpretation of the data is thorough. Highlighting the misconceptions toward cannabis-based products for recreational versus medical use is timely, a relevant question and will help reduce the risks of pain patients in using products that contain recreational amounts of THC.

**Comments:**

Limitation of the study: the manuscript clearly mentions that the information collected was from dispensaries online and discusses specifies limitations. One aspect that should be emphasized more is that, at least in WA state, the values of THC and CBD on cannabis product labels are not always accurate.

**Response:**

We thank the reviewer for pointing out the limitations of our study. In accordance to this comment, our original discussion has a paragraph in which we address the inaccuracy of THC and CBD content in labels and in the methods used to measure these cannabinoids. We have added additional comments related to this and how this poses a limitation to our study. The changes are in blue font. The new text reads as follows:

“Although consistent with other cannabinoid concentrations reported throughout the literature, no universal standards for laboratory testing exist and previous studies have demonstrated the tendency for some laboratories to consistently report higher cannabinoid concentrations than their counterparts [28], which might contribute to the lack of accuracy in THC or CBD content in the labels of cannabis products [47]. Regardless of the method or laboratory used to measure THC content, the average THC concentration in all cases has been reported to be above 15% since 2014 [28]. Thus, the THC content marketed online is consistent with reports in which THC was actually measured, suggesting a high level of concordance of our data with the existing literature, and suggesting the degree of inaccuracy of our data is similar to these studies. In addition to discrepancies between laboratories, a recent study demonstrated that CBD products advertise an accurate CBD concentration only 31% of the time and unlabeled THC was detected in about 21% of products [47]. Such results suggest a need for regulations that improve transparency in regard to the location and methods by which each product was tested.

The inability to confirm the accuracy of reported THC and CBD is undoubtedly a limitation of this study. However, the concentrations reported in this study are representative of what a consumer would assess and therefore hold significant implications. The accuracy, or lack thereof, of labeled cannabinoid content of legal cannabis products poses a concern over patient’s misconceptions regarding the potency of products they consume.”

**Comments:**

Another limitation that should be mentioned is that some states allow patients to grow their medical cannabis and thus this population is not included in this study.

**Response:**

We have including the following phrase (blue font) in the discussion. The third paragraph now reads as follows:

“We did not map concentrations in all dispensaries in all U.S. states where cannabis is legalized for either medical treatment of pain or recreational use; however, our study covers the two regions where most states have such programs. In contrast to smaller yet well-designed studies, our study covers Western and Northeastern regions from the U.S. and 8,505 cannabis chemotypes/products across 653 dispensaries [27]. Therefore, our results are likely a fair representation of the potency of online products offered in dispensaries across the U.S. It is challenging to generalize our findings to states not included in our study given the difference in legal requirements, size of state, population, and other demographics may drive the production and dispensing patterns in each state. Furthermore, state legal requirements differ in regard to home cultivation of cannabis, a population that was not represented in this study yet may also drive demand and further influence the production and dispensing in each state. However, our findings appear to be consistent and surprisingly uniform within and between the Western and Northeastern regions, which comprise very diverse geographic and demographic size, and differ widely in their cannabis program legal requirements. Our study indicates that both recreational and medicinal programs in various and diverse regions of the country are contributing to and reflecting the national trend towards increasing potency of cannabis.”

**Comments:**

“A total of 8,505 cannabis strains across 653 dispensaries were sampled”. The specifics of what constitutes a “strain” is needs to be clarified. This reviewer was not aware that there were so many strains. Adding a reference here would help the reader better understand the extent of the diversity in strains.

**Response:**

We appreciate this comment. In an effort to reflect what we found in online information, we kept the terminology used in dispensaries’ websites in our manuscript. We recognize that this is not correct, and we have edited the manuscript accordingly. We now do not use “strain”, instead we use “chemotype” or simply “product”. We consider both terms to be more scientifically sound and accurate. Similarly, we do not use the classification “Sativa” or “Indica”, since these are also not scientifically accurate. As suggested by experts in the field (Piomelli D and Russo EB, Cannabis and Cannabinoid Research 2016), we use instead THC-rich, CBD-rich, and mixed THC/CBD. For example, the first paragraph of the Data Collection sub-section in the Methods section now reads as follows:

“Cannabinoid data was collected for all chemotypes of THC-rich, CBD-rich, and mixed THC/CBD flowers and pre-rolls. It is worth mentioning that the dispensary websites referred to the advertised products as “strains” and classified such products as “Sativa”, “Indica” or “Hybrid”.”

**Comments:**

“a decline in the number of opioid prescriptions”. Several recent studies show that this is not always the case, depending on how the results are analyzed and whether recent years data are included or not. These studies concluding the opposite view, that legalization has not affected opioid use, need to be mentioned here.

**Response:**

We appreciate Reviewer 3 bringing this to our attention. We agree, our previous comment on the topic was very succinct and could have given the impression of bias. We also appreciate the opportunity to clarify this point. We have re-phrased our previous statement and added a comment and reference about conflicting evidence. The new language reads as follows in the introduction (first paragraph):

“While it is challenging to determine a cause and effect relationship between the implementation of cannabis legislation and a decline in opioid prescribing practices given the concurrent rise of legislation aimed at restricting and monitoring opioid prescribing, states with legalized medical cannabis programs have witnessed a decline in the number of opioid prescriptions and prescribed doses, particularly among younger cohorts and for schedule III opioids[1-4]. Interestingly, recreational cannabis legislation has not demonstrated a significant impact on opioid prescribing in privately insured adults [5].”

**Comments:**

“accessible in a non-uniformly regulated market in contrast to other countries”. This reads as a bold statement. More details will help the reader better understand the context of this statement.

**Response:**

We apologize for not being sufficiently clear in this point and appreciate the opportunity to provide more background information to make this statement more accurate. We added more context to this point. The second paragraph in the introduction reads as follows:

“Thus, the U.S. represents a largely populated geographical area in which cannabis is becoming rapidly legal and accessible in a non-uniformly regulated market in contrast to other countries such as Uruguay, which has slowly implemented it’s cannabis policies in order to address challenges and insure the safe and successful implementation of a nation-wide cannabis program.”

**Comments:**

“Alternatively, CBD ameliorates the euphoric effects and counteracts the unintended adverse effects of THC (14)”. This sentence is confusing. How can CBD ameliorate the euphoria of something that is already euphoric, and what are the side effects of THC that are referred to here?

**Response:**

We apologize for not being clear in this statement. We have reworded this sentence to make it more accurate. Now the text is as follows:

“Alternatively, CBD has demonstrated the potential to ameliorate the psychotic- and anxiety-inducing effects of THC [23, 24] and may mitigate its memory-impairing effects as well [25, 26].”

**Comments:**

“This study will also evaluate the appropriateness of available strains for medicinal use”. What level of diligence is dedicated to validating the strains? Where there any genetic profiling done? Some language should be added here in the manuscript.

**Response:**

Thanks for opportunity to clarify this point. We have addressed the limitations of our study in a previous comment and elsewhere in our manuscript as suggested by this reviewer. Based on those changes we re-phrased this statement by adding that we based the appropriateness concept on the advertised potency of products. The new phrase reads as follows:

“This study will also evaluate the appropriateness of available products for medicinal use based on the advertised potency and determine whether a difference exists between the strength of cannabis offered by medical and recreational programs.”

**Comments:**

The resolution of the various figures and graphs was low in this reviewers PDF file and should be improved.

**Response:**

We apologize for the low quality of the original images (they were 300 dpi). We now include higher resolution images, i.e. 600 dpi.

6. PLOS authors have the option to publish the peer review history of their article ([what does this mean?](https://journals.plos.org/plosone/s/editorial-and-peer-review-process#loc-peer-review-history)). If published, this will include your full peer review and any attached files.


If you choose “no”, your identity will remain anonymous but your review may still be made public.


**Do you want your identity to be public for this peer review?** For information about this choice, including consent withdrawal, please see our [Privacy Policy](https://www.plos.org/privacy-policy).

Reviewer #1: No

Reviewer #2: Yes: Adrianne Wilson-Poe

Reviewer #3: No
